# OPTN recruitment to a Golgi-proximal compartment regulates immune signalling and cytokine secretion

**DOI:** 10.1242/jcs.239822

**Published:** 2020-06-15

**Authors:** Thomas O'Loughlin, Antonina J. Kruppa, Andre L. R. Ribeiro, James R. Edgar, Abdulaziz Ghannam, Andrew M. Smith, Folma Buss

**Affiliations:** 1 Cambridge Institute for Medical Research, The Keith Peters Building, University of Cambridge, Hills Road, Cambridge CB2 0XY, UK; 2 Helen Diller Family Comprehensive Cancer Center, San Francisco, CA 94158, USA; 3 Microbial Diseases, Eastman Dental Institute, University College London, London WC1X 8LD, UK; 4 Department of Oral and Maxillofacial Surgery, University Centre of Pará, Belém, Brazil

**Keywords:** OPTN, BioID, Functional proteomics, NF-κB, TBK1, IFN, Linear ubiquitin, LUBAC, ATG9A

## Abstract

Optineurin (OPTN) is a multifunctional protein involved in autophagy and secretion, as well as nuclear factor κB (NF-κB) and IRF3 signalling, and *OPTN* mutations are associated with several human diseases. Here, we show that, in response to viral RNA, OPTN translocates to foci in the perinuclear region, where it negatively regulates NF-κB and IRF3 signalling pathways and downstream pro-inflammatory cytokine secretion. These OPTN foci consist of a tight cluster of small membrane vesicles, which are positive for ATG9A. Disease mutations in OPTN linked to primary open-angle glaucoma (POAG) cause aberrant foci formation in the absence of stimuli, which correlates with the ability of OPTN to inhibit signalling. By using proximity labelling proteomics, we identify the linear ubiquitin assembly complex (LUBAC), CYLD and TBK1 as part of the OPTN interactome and show that these proteins are recruited to this OPTN-positive perinuclear compartment. Our work uncovers a crucial role for OPTN in dampening NF-κB and IRF3 signalling through the sequestration of LUBAC and other positive regulators in this viral RNA-induced compartment, leading to altered pro-inflammatory cytokine secretion.

## Abstract (precis)

**Summary:** Disease-associated OPTN mutations impact the formation of a viral RNA-induced compartment, which is important for regulating NF-κB and IRF3 signalling and pro-inflammatory cytokine secretion.

## INTRODUCTION

Pathogen associated molecular patterns (PAMPs) are recognised by pattern recognition receptors (PRRs), such as Toll-like receptors (TLRs), and trigger a range of adaptive and innate immune responses in the host (Takeuchi and Akira, 2010). For example, activation of TLR3 or RIG-I (also known as DDX58) by double-stranded viral RNA activates signalling cascades culminating in the activation of transcription factors including nuclear factor κB (NF-κB) and IRF3 and gene expression programs composed of pro-inflammatory cytokines (e.g. IL6) and interferons (IFNs), respectively (Alexopoulou et al., 2001). Optineurin (OPTN) appears to be a key protein in a range of pathways downstream of TLR3, participating in the innate immune response through the secretion of cytokines, acting as a selective autophagy receptor, and regulating both NF-κB and IRF3 signalling (Slowicka et al., 2016).

NF-κB signalling centres around the NF-κB transcription factor complex which, under non-stimulated conditions, is inhibited through binding to IκB proteins. In response to stimuli, such as TLR3 or RIG-I ligation, the pathway is switched on leading to activation of the IKK complex, composed of two kinase subunits (IKKα and IKKβ; also known as CHUK and IKBKB, respectively) and a regulatory subunit IKKγ [IKBKG; also known as NF-κB essential modulator (NEMO)], which phosphorylates IκB proteins and triggers their subsequent degradation. This degradation releases the NF-κB complex, allowing it to translocate to the nucleus and induce expression of numerous target genes (Perkins, 2007). An additional critical step in this pathway is the linear M1-linked ubiquitylation of NEMO and receptor-interacting protein kinase 1 (RIPK1) by the linear ubiquitin assembly complex (LUBAC), which consists of HOIP (RNF31), HOIL1 (RBCK1) and SHARPIN (Gerlach et al., 2011; Kirisako et al., 2006; Tokunaga et al., 2009, 2011). These linear ubiquitin chains can then function as scaffolds to recruit the IKK complex through the ubiquitin binding in ABIN and NEMO (UBAN) domain of the IKK subunit NEMO (Fujita et al., 2014; Rahighi et al., 2009; Wagner et al., 2008). OPTN is highly similar to NEMO, with around 52% sequence similarity, and shares its linear ubiquitin-binding UBAN domain (Schwamborn et al., 2000; Wagner et al., 2008). However, unlike NEMO, OPTN cannot bind to IKKα or IKKβ and therefore cannot rescue NF-κB activity in NEMO-deficient cells (Schwamborn et al., 2000). Instead, OPTN appears to antagonise NEMO function by competitively binding to ubiquitylated RIPK1 and can thereby inhibit TNFα-induced NF-κB activation (Zhu et al., 2007). In addition, OPTN interacts with CYLD, a deubiquitylase (DUB) for linear and K63 ubiquitin chains, which is able to negatively regulate NF-κB signalling via the deubiquitylation of a range of NF-κB signalling proteins including NEMO and RIPK1 (Lork et al., 2017; Nagabhushana et al., 2011).

Alternatively, OPTN can bind to the IKK-related kinase TBK1 or the E3 ligase TRAF3 to regulate IRF3 activity (Mankouri et al., 2010; Morton et al., 2008). A complex composed of TBK1 and IKKε is activated via TRAF3 downstream of PRRs, such as TLR3 or RIG-I. Once active, the TBK1–IKKε complex can phosphorylate its substrate, IRF3, which subsequently dimerises and translocates to the nucleus to induce expression of target genes such as type I IFNs (IFNs). Through its interactions with both TBK1 and TRAF3, OPTN appears to attenuate IFN-β production (Mankouri et al., 2010).

An increasing number of perturbations in *OPTN* gene function have been linked to diseases including primary open-angle glaucoma (POAG), amyotrophic lateral sclerosis (ALS), Paget's disease of bone (PDB) and Crohn's disease (CD) (Albagha et al., 2010; Maruyama et al., 2010; Rezaie et al., 2002; Smith et al., 2015). A common feature of the role of OPTN in these diseases appears to be aberrant NF-κB signalling or cytokine secretion profiles. Many ALS mutants show a loss of OPTN-mediated NF-κB suppression (Nakazawa et al., 2016), deficiencies in OPTN expression increase NF-κB activity and susceptibility to PDB (Obaid et al., 2015) and a subset of CD patients with reduced OPTN expression display impaired secretion of TNF-α, IL6 and IFN-γ (Smith et al., 2015).

In this study, we address the role of OPTN in innate immune signalling and cytokine secretion, and the mechanism by which perturbation of OPTN function in these processes may contribute to human inflammatory disease. We use a retinal pigment epithelial (RPE) cell model, which is relevant to the role of OPTN in the pathogenesis of POAG, and show these cells respond to TLR3 and RIG-I ligands, leading to upregulation of OPTN and its translocation to perinuclear foci. Our ultrastructural analysis of these foci by correlative light and electron microscopy reveals that this compartment consists of a tight cluster of small vesicles, which appear positive for the autophagy protein ATG9A. This multispanning membrane protein is present at the Golgi complex and in clusters of small 30–40 nm vesicles, which are often found in close proximity to autophagosomes, but do not appear to be incorporated into the growing phagophore (Orsi et al., 2012; Young et al., 2006). We demonstrate that wild-type or mutant variants of OPTN show variable recruitment to this vesicle cluster, which correlates with the ability to negatively regulate NF-κB and IRF3 signalling and therefore cytokine secretion. Using proximity-dependent proteomics (BioID) to characterise this compartment, we identify novel OPTN-interacting proteins including IFT74, IFI35, a phosphoinositide phosphatase complex (MTMR6–MTMR9) and the LUBAC, with the latter being recruited to OPTN-positive foci upon TLR3 ligation. Our data suggest that OPTN can inhibit the innate immune response through sequestering key components of NF-κB and IRF3 signalling pathways in a novel perinuclear compartment. Disease-associated OPTN mutations impact on the formation of the perinuclear compartment and result in hypo- or hyper-activation of the immune response, which could potentially drive the development of a number of human diseases.

## RESULTS

### RPE cells exhibit a robust response to double-stranded RNA

RPE cells perform a number of support functions in the inner eye including the secretion of signalling molecules and the maintenance of the immune privileged environment through communication with the immune system (Detrick and Hooks, 2010). Previous reports have demonstrated that RPE cells express a number of TLRs including the viral RNA receptor TLR3 (Kumar et al., 2004). OPTN mutations have been implicated in POAG (Kumar et al., 2016; Rezaie et al., 2002), making the RPE cell line a relevant tool to study OPTN function in this disease. Furthermore, the proposed roles for OPTN in anti-viral immunity and TLR3 signalling led us to investigate the utility of this cell line as a tractable human model for OPTN function in these pathways.

**Fig. 1.**
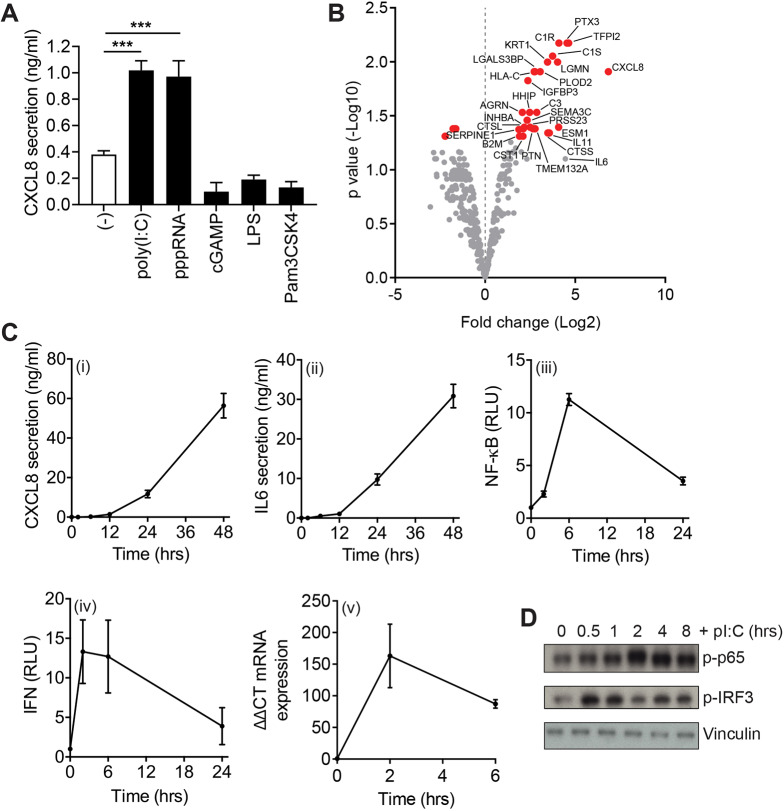
**RPE cells show a robust TLR3 and RIG-I response.** (A) CXCL8 secretion from RPE cells stimulated with the indicated ligands. Bars depict mean±s.e.m. of ≥*n*=4 independent experiments. ***P<0.001 (one-way ANOVA and a Bonferroni post-hoc test). (B) Volcano plot of fold change versus adjusted *P*-value from SILAC secretome experiments (*n*=3). Red points show a significant *P*-value change (*P*<0.05) in the poly(I:C)-stimulated versus unstimulated condition. Significantly enriched and other notable proteins are labelled. (C) Poly(I:C)-induced CXCL8 secretion (i), IL6 secretion (ii), NF-κB luciferase reporter activity (iii), IFNα/β secretion (iv) and IFN-β mRNA expression (v) in RPE cells over the time courses indicated. Bars depict mean±s.e.m. of *n*=4 (i, ii, iv) or *n*=3 (iii, v) experiments. RLU, relative light units. (D) Immunoblot analysis of lysates from RPE cells stimulated with poly(I:C) for the indicated times and probed for p-p65(Ser536), p-IRF3 and vinculin (loading control). The same blot was stripped and re-probed with antibodies to p-TBK1 in Fig. 3A.

RPE cells were stimulated with a range of PAMPs and the immune response determined through the quantification of CXCL8 secretion. Of all the PAMPs tested, only poly(I:C) and 5′-triphosphate double-stranded (ds)RNA (pppRNA) induced significant CXCL8 secretion, consistent with the expression and activation of TLR3 and RIG-I in RPE cells (Fig. 1A). Lipopolysaccharide (LPS), Pam3CSK4 and 2′,3′-cGAMP (cGAMP) were unable to elicit the release of CXCL8 from RPE cells, illustrating a lack of activation downstream of TLR4, TLR2 and STING (also known as STING1). To determine the complete secretory response of RPE cells downstream of poly(I:C) stimulation, we analysed conditioned medium from unstimulated or poly(I:C)-stimulated RPE cells using quantitative SILAC mass spectrometry. These experiments identified 380 proteins in the conditioned medium with 26 showing significant (*P*<0.05) upregulation (Fig. 1B; Table S1). Among the upregulated proteins were well-known pro-inflammatory cytokines, such as CXCL8 and, to a lesser extent, IL6 (Fig. 1B). We validated this data by ELISA and found poly(I:C) stimulation resulted in the induction of both CXCL8 and IL6 protein secretion (Fig. 1Ci,ii). To assess the contribution of NF-κB signalling in regulation of cytokine secretion, we generated an RPE cell line expressing a NF-κB luciferase reporter. We found that stimulating these cells with poly(I:C) induced NF-κB promoter activity and a similar elevation in phosphorylated p65 (also known as RELA; an NF-κB subunit) was observed using immunoblot analysis (Fig. 1Ciii,D). Although no IFNs were detected in the proteomics datasets, we predicted that IRF3 signalling would also be active downstream of TLR3 (Doyle et al., 2002). Indeed, upon poly(I:C) stimulation, we observed a rapid phosphorylation of IRF3, an elevation in IFN-β (*IFNB1*) mRNA levels, and could detect IFNs in the supernatant at 2 h post-stimulation (Fig. 1Civ,v,D).

### OPTN translocates to a novel perinuclear compartment in response to double-stranded RNA

**Fig. 2.**
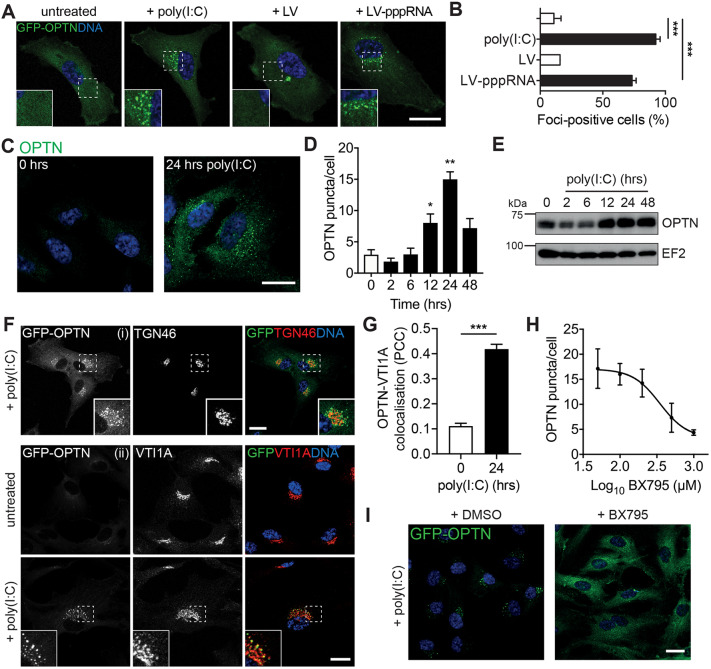
**OPTN is recruited to a novel compartment in response to single- and double-stranded viral RNA.** (A) Confocal microscopy images of RPE cells stably expressing GFP–OPTN (green) and treated with vehicle, poly(I:C), Lyovec (LV) or pppRNA transfected with LV for 24 h. Cells were stained with Hoechst to label DNA (blue). Insets show magnified views of regions indicated by dashed boxes. Scale bar: 20 µm. (B) Percentage of GFP–OPTN cells containing foci after treatment. >100 cells were manually counted per condition from ≥10 randomly selected fields of view. Bars represent the mean±s.e.m. of *n*=3 independent experiments (except for LV where *n*=2). ***P<0.001 (repeated measures ANOVA and Bonferroni post-hoc test). (C) Confocal microscopy images of RPE cells stimulated with poly(I:C) for 0 and 24 h. Cells were immunostained with an anti-OPTN antibody (green) and DNA was visualised with Hoechst (blue). Scale bar: 20 µm. (D) Foci count per GFP–OPTN cell after treatment with poly(I:C) for the indicated times. Bars represent mean±s.e.m. of *n*=3 independent experiments. *P<0.05; **P<0.01 vs 0 h time point (repeated measures ANOVA and a Bonferroni post-hoc test). (E) Immunoblot analysis of lysates from RPE cells stimulated with poly(I:C) for indicated times and probed Insets show magnified views of regions indicated by dashed boxes. with OPTN and EF2 (loading control) antibodies. (F) Confocal microscopy images of RPE cells stably expressing GFP–OPTN (green) and treated with poly(I:C) where specified. Cells were immunostained with an antibody against TGN46 (i) and VTI1A (ii) (red). Insets show magnified views of regions indicated by dashed boxes. Scale bars: 20 µm. (G) Pearson's correlation coefficient (PCC) calculated for GFP–OPTN versus VTI1A after treatment with poly(I:C) for 0 and 24 h. Bars represent the mean±s.e.m. of *n*=3 independent experiments. Cells were quantified from ≥20 randomly selected fields of view (one cell/image). ***P<0.001 (two-sample *t*-test). (H) Dose–response curve of foci count per GFP–OPTN cell after treatment with poly(I:C) for 24 h in combination with the indicated dose of BX795. Points represent mean±s.e.m. of *n*=3 experiments. (I) Confocal microscopy images of RPE cells stably expressing GFP–OPTN (green) and treated with poly(I:C) for 24 h in combination with DMSO (left panel) or BX795 (right panel). Scale bar: 20 µm.

Transient overexpression of OPTN triggers the formation of Golgi-proximal foci (Mao et al., 2017; Maruyama et al., 2010; Nagabhushana et al., 2010; Park et al., 2006, 2010; Shen et al., 2011; Turturro et al., 2014; Ying et al., 2010), which have been postulated to be aggresomes (Mao et al., 2017) or organelles participating in post-Golgi membrane trafficking and the maintenance of Golgi integrity (Nagabhushana et al., 2010; Park et al., 2006, 2010). We observed that stably expressed GFP–OPTN was predominantly cytosolic in resting RPE cells but, strikingly, translocated to perinuclear foci after stimulation with both poly(I:C) or pppRNA (Fig. 2A,B), but not with other PAMPs, such as LPS, cGAMP or Pam3CSK4 (Fig. S1A). Similarly, endogenous OPTN was recruited from a diffuse cytosolic pool to bright foci in the perinuclear region in poly(I:C)-stimulated RPE cells (Fig. 2C). We assessed the rate of formation of this compartment and discovered that the foci began to form beyond 2 h post-stimulation before peaking at ∼24 h (Fig. 2D). *OPTN* gene expression is regulated through NF-κB signalling (Sudhakar et al., 2009) and increases upon TLR3 activation by poly(I:C) or viral infection (Mankouri et al., 2010). Similarly, we observed that expression of OPTN is markedly upregulated in response to poly(I:C) stimulation in RPE cells with kinetics similar to foci formation (Fig. 2E). This data suggests that elevated OPTN expression triggers its accumulation into perinuclear foci.

To further analyse the nature of this perinuclear OPTN-positive compartment, we labelled GFP–OPTN-expressing cells with a variety of organelle markers. The foci showed very little overlap with markers of the endocytic pathway, including EEA1 and LAMP1 (Fig. S1Bi,ii). Notably, the foci could be observed in close proximity to, but only showed partial colocalisation with the trans-Golgi marker TGN46 (also known as TGOLN2), the cation-independent mannose-6-phosphate receptor (CIMPR, also known as IGF2R) or the autophagosomal membrane marker LC3 (also known as MAP1LC3B; Fig. 2Fi; Fig. S1Biii,iv). Further observations indicated strong colocalisation with the OPTN-binding partner MYO6 (Fig. S1C) and with the Golgi SNARE VTI1A (Fig. 2Fii,G), suggesting some continuation with the Golgi complex. Depletion of MYO6 by siRNA had no effect on the formation of the foci indicating that the recruitment of OPTN to these structures and the formation of the foci was not dependent on MYO6 (Fig. S1D).

### TBK1 activity is necessary for OPTN recruitment to foci but not their long-term stability

**Fig. 3.**
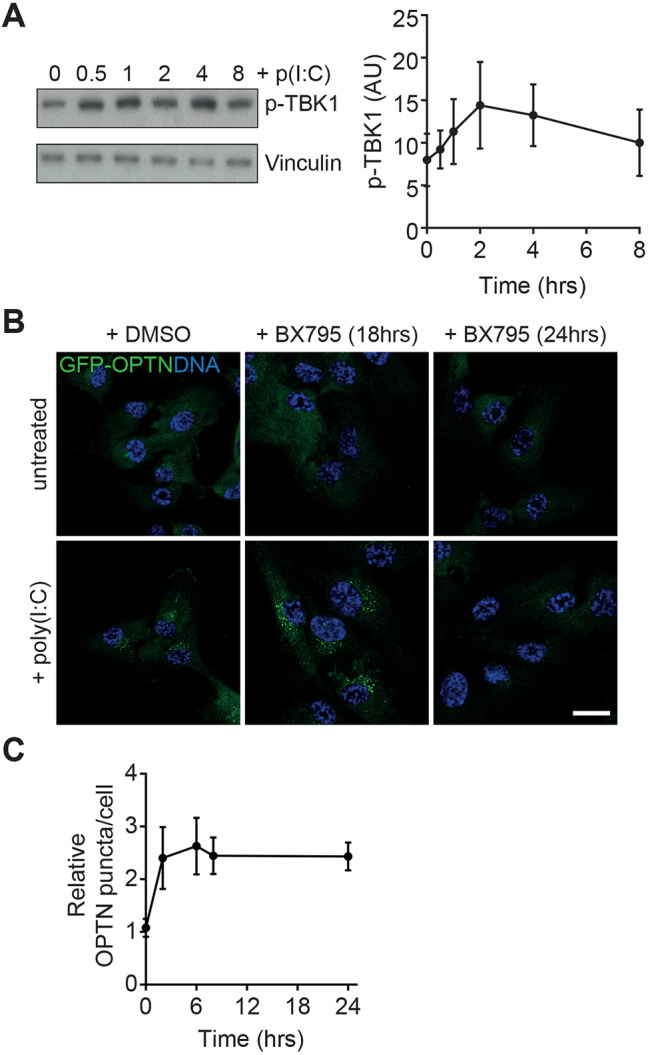
**TBK1 inhibition perturbs foci formation.** (A) Left, immunoblot analysis of lysates from RPE cells stimulated with poly(I:C) for the indicated times; the immunoblot shown in Fig. 1D was stripped and re-probed with p-TBK1 antibodies. The same vinculin blot was therefore also used as loading control (see Fig. 1D). Right, graph depicting gel band density analysis for p-TBK1. Points represent mean±s.e.m. of *n*=3 experiments. (B) Confocal microscopy images of RPE cells stably expressing GFP–OPTN (green) and treated with vehicle (top row) or poly(I:C) for 24 h (bottom row). Cells were simultaneously treated with DMSO or BX795 for 18 h (added after 6 h) or 24 h (added after 0 h). DNA was visualised with Hoechst (blue). Scale bar: 20 µm. (C) Relative foci counts per GFP–OPTN cell after treatment with poly(I:C) for 24 h combined with BX795 addition after the indicated times. Points represent mean±s.e.m. of *n*=3 independent experiments.

Given the well-established role of TBK1 and OPTN in the antiviral response (Pourcelot et al., 2016), we next assessed the role of TBK1 in foci formation. TBK1 activity measured through the increase in its phosphorylation (p-TBK1), was evident 30 min post-TLR3 stimulation and returned to baseline levels after 8 h (Fig. 3A). Using a specific inhibitor of TBK1, BX795, OPTN foci formation could be abolished in a dose-dependent fashion downstream of TLR3 activation (Fig. 2H,I). Interestingly, addition of BX795 at 6 h after poly(I:C) stimulation did not influence foci formation (Fig. 3B,C), which indicates that TBK1 kinase activity is required for initiation of the foci but is dispensable for the subsequent maintenance of the structure.

### OPTN disease mutants show perturbed foci formation

**Fig. 4.**
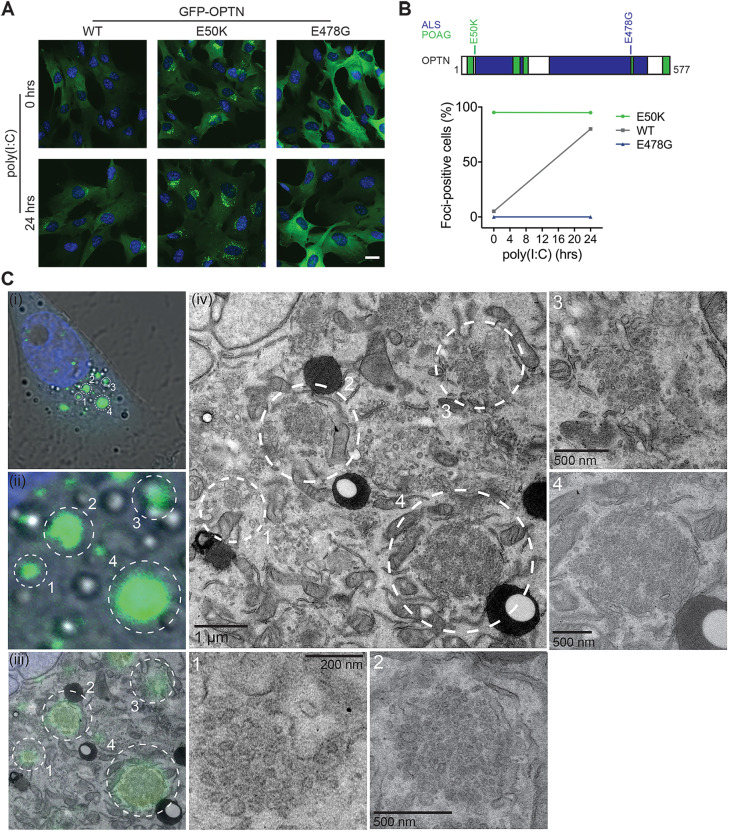
**OPTN disease mutants promote aberrant foci formation.** (A) Widefield microscopy images of RPE cells stably expressing GFP–OPTN wild-type (WT), E50K and E478G (green) and treated for 0 h or 24 h with poly(I:C). Cells were stained with Hoechst to label DNA (blue). Scale bar: 20 µm. (B) Top, schematic cartoon of OPTN domain structure with mutations highlighted. Bottom, graph depicting the mean±s.e.m. percentage of GFP–OPTN cells containing foci after 0 or 24 h of poly(I:C) treatment from *n*=3 independent experiments; >100 cells were manually counted per condition from ≥10 randomly selected fields of view. Error bars do not extend beyond the data points plotted due to the low values of s.e.m. for this experiment. (C) Correlative light electron microscopy (CLEM) micrographs of RPE cells stably expressing GFP–OPTN E50K. (i) Confocal microscopy image of a cell and (ii) magnification of four GFP-positive foci highlighted by the circled regions 1, 2, 3 and 4. (iii) Electron micrograph with confocal microscopy image of GFP-positive foci superimposed. (iv) Electron micrograph of foci-positive region. Four GFP-positive foci are highlighted by the circled regions 1, 2, 3 and 4, and are magnified in the corresponding panels 1–4.

Previous work has linked the OPTN E50K mutant to POAG and has shown that OPTN overexpression causes the formation of large perinuclear foci in cells (Nagabhushana et al., 2010; Park et al., 2006, 2010; Rezaie et al., 2002; Turturro et al., 2014). Conversely, the E478G mutation, which is linked to ALS, appears to lack this capacity (Maruyama et al., 2010; Turturro et al., 2014). We predicted that these mutants might show a perturbed ability to form foci in response to poly(I:C) stimulation. Strikingly, ∼95% of RPE cells expressing GFP–OPTN E50K exhibited a constitutive formation of this compartment even in the absence of stimuli, compared to ∼5% of cells expressing wild-type GFP–OPTN (Fig. 4A,B). TLR3 stimulation resulted in ∼80% of wild-type GFP-OPTN expressing cells making foci, whereas stimulation had minimal effect on the GFP–OPTN E50K cells that retained foci in ∼95% of cells. By contrast, cells expressing GFP–OPTN E478G were completely unable to generate foci even after 24 h of poly(I:C) stimulation (Fig. 4A,B).

Interestingly, although foci formation is triggered by TLR3-stimulation the receptor was not recruited into OPTN foci, suggesting that this compartment is distinct from the route of receptor trafficking (Fig. S2A). Furthermore, perturbation of TLR3 expression using CRISPR interference (CRISPRi) largely blocked poly(I:C)-induced OPTN foci formation, indicating that this phenotype is dependent on TLR3 receptor-driven signalling (Fig. S2B–D).

To visualise the nature and further define the composition of the OPTN-positive compartment, we performed correlative light electron microscopy (CLEM) on foci generated by the OPTN E50K mutant. Cells were first imaged by confocal microscopy to determine the localisation of the GFP–OPTN E50K-positive foci and then processed for electron microscopy. CLEM images showed that the foci were composed of tightly packed small membrane vesicles contained within a spherical area void of any further delimiting membrane (Fig. 4C). As aggresomes are typically membrane-less and electron-dense structures (Kopito, 2000), our data would appear to rule out the possibility that OPTN foci are simply protein inclusions, but are clearly a membranous compartment consisting of a cluster of small vesicles of uniform size.

### OPTN-positive vesicle clusters colocalise with ATG9A

**Fig. 5.**
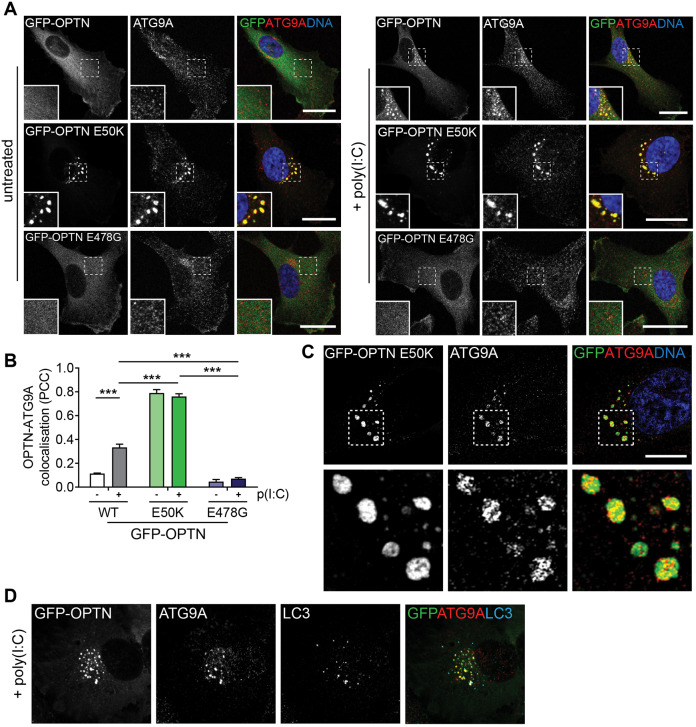
**OPTN-positive vesicle clusters colocalise with ATG9A.** (A) Confocal microscopy images of RPE cells stably expressing GFP–OPTN, GFP–OPTN E50K or GFP–OPTN E478G (green) and treated with poly(I:C) for 0 h (left) or 24 h (right). Cells were immunostained with an anti-ATG9A antibody (red) and Hoechst to label DNA (blue). Insets show magnified views of regions indicated by dashed boxes. Scale bars: 20 µm. (B) Pearson's correlation coefficient (PCC) calculated for GFP–OPTN (WT, E50K or E478G) versus ATG9A after treatment with poly(I:C) for 0 and 24 h. Bars represent the mean±s.e.m. of *n*=3 independent experiments. Cells were quantified from ≥10 randomly selected fields of view. ***P<0.001 (repeated measures ANOVA and a Bonferroni post-hoc test). (C) Structured illumination microscopy image of RPE cells stably expressing GFP–OPTN E50K (green), immunostained with ATG9A antibody (red) and Hoechst to label DNA (blue). Scale bar: 10 µm. Lower panels are magnifications of the regions highlighted above. (D) Confocal microscopy images of RPE cells stably expressing GFP–OPTN (green) and treated with poly(I:C) for 24 h. Cells were immunostained with an ATG9A antibody (red) and LC3 antibody (blue). Scale bar: 15 µm.

ATG9A has been implicated in the innate immune response to cytosolic DNA, where it regulates the assembly of STING and TBK1 on a vesicular Golgi-associated perinuclear compartment (Saitoh et al., 2009). To determine whether the cellular response to viral RNA involves a similar ATG9A compartment, we determined whether the OPTN-positive vesicles colocalise with ATG9A. In unstimulated RPE cells, ATG9A is present at the Golgi complex, however, after poly(I:C) stimulation the newly formed GFP-OPTN foci are positive for ATG9A (Fig. 5A–C). Observations of OPTN mutants revealed that GFP–OPTN E50K appeared to trap ATG9A on Golgi-proximal foci even in the absence of stimuli, while GFP–OPTN E478G failed to colocalise with ATG9A even after stimulation (Fig. 5A–C). High-resolution microscopy reveals the presence of distinct ATG9A-containing vesicle clusters appearing to decorate the OPTN-positive foci. Interestingly, the OPTN-positive foci are occasionally in close proximity but show only limited overlap with LC3-positive autophagosomes (Fig. S1B). Furthermore, the poly(I:C) induced ATG9A-positive foci show very little overlap with LC3-positive membranes, confirming previous data that the ATG9A-containing vesicles might interact with but do not appear to be incorporated into the growing phagophore (Orsi et al., 2012; Karanasios et al., 2016) (Fig. 5D).

### BioID reveals novel OPTN partners and foci proteins

**Fig. 6.**
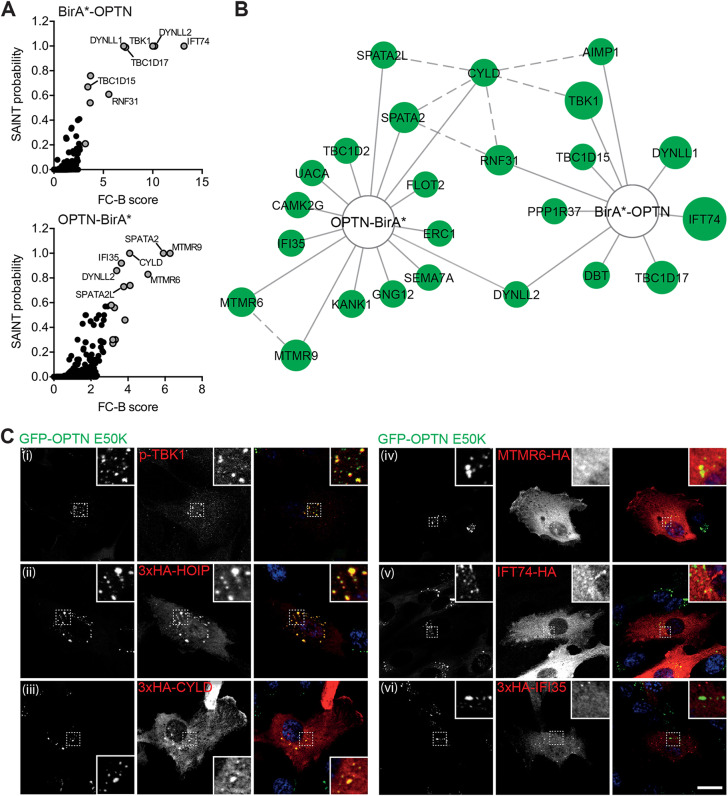
**OPTN BioID reveals novel partners and proteins localised to foci.** (A) Graphs depicting significance analysis of interactome (SAINT) probability and fold change (FC-B) scores for BirA*–OPTN (top) and OPTN–BirA* (bottom) pulldown experiments. Selected high-confidence OPTN interactors are labelled. (B) Network diagram of high-confidence OPTN interactors identified by BioID. Node size corresponds to FC-B score (higher confidence=larger node). Solid lines indicate interactions identified in this study, and dashed lines interactions imported from publicly available protein–protein interaction databases. (C) Confocal microscopy images of RPE cells stably expressing GFP–OPTN E50K (green) and immunostained with an anti-p-TBK11 (i; red) or anti-HA antibody (ii–vi; red). Insets show magnified views of regions indicated by dashed boxes. Scale bar: 20 µm.

To gain further insight into both OPTN function and the composition of the foci, we determined the OPTN interactome using *in situ* proximity labelling. We generated RPE stable cell lines expressing full-length OPTN tagged at the N- or C-terminus with the promiscuous biotin ligase, BirA R118G (BirA*). Expression of the BirA*–OPTN or OPTN–BirA* fusion proteins was verified by immunoblotting and the localisation assessed by immunofluorescence (Fig. S3A,B). After labelling with biotin overnight, we performed streptavidin pulldowns and identified the enriched proteins by mass spectrometry. Replicates were analysed against a bank of five BirA*-only RPE1 control pulldowns using the online tool at http://crapome.org/ and using a threshold fold change (FC)-B score of ≥3, we identified 25 significantly enriched proteins (Tables S2, S3) (Mellacheruvu et al., 2013). Among the proteins we identified, were a number of known OPTN-interacting proteins and complexes such as TBK1, CYLD, TBC1D17 and the LUBAC component HOIP (also known as RNF31) in addition to novel putative interactors such as the myotubularin-related (MTMR) lipid phosphatase complex components MTMR6 and MTMR 9, intraflagellar transport 74 (IFT74) and interferon-induced protein 35 (IFI35) (Fig. 6A,B).

We screened a selection of these candidates for their ability to localise to GFP–OPTN E50K-induced foci (Fig. 6C) including p-TBK1, which has been shown previously to colocalise with OPTN (Fig. 6Ci) (Mankouri et al., 2010). Interestingly, the E3 ligase, HOIP, as well as the DUB, CYLD, both showed colocalisation on OPTN foci, although CYLD only showed recruitment in a small subpopulation of cells (Fig. 6Cii,iii). In contrast, MTMR6, IFT74 and IFI35 showed little recruitment to OPTN foci (Fig. 6Civ–vi), and might interact with OPTN within other cellular pathways such as autophagy.

### The LUBAC is recruited to OPTN foci

NEMO interacts with, and is linearly ubiquitylated by, LUBAC to induce the activation of the IKK complex (Rahighi et al., 2009; Tokunaga et al., 2009). OPTN also binds LUBAC components HOIP and HOIL1 and regulates the interaction of RIPK1 and NEMO with the TNF receptor (TNFR) complex in response to TNF-α (Nakazawa et al., 2016). Our BioID experiments are consistent with the concept that HOIP interacts with OPTN but also indicate a possible co-recruitment to OPTN foci. Furthermore, the potential cooperation of HOIP and OPTN in TLR3 signalling remains unexplored.

**Fig. 7.**
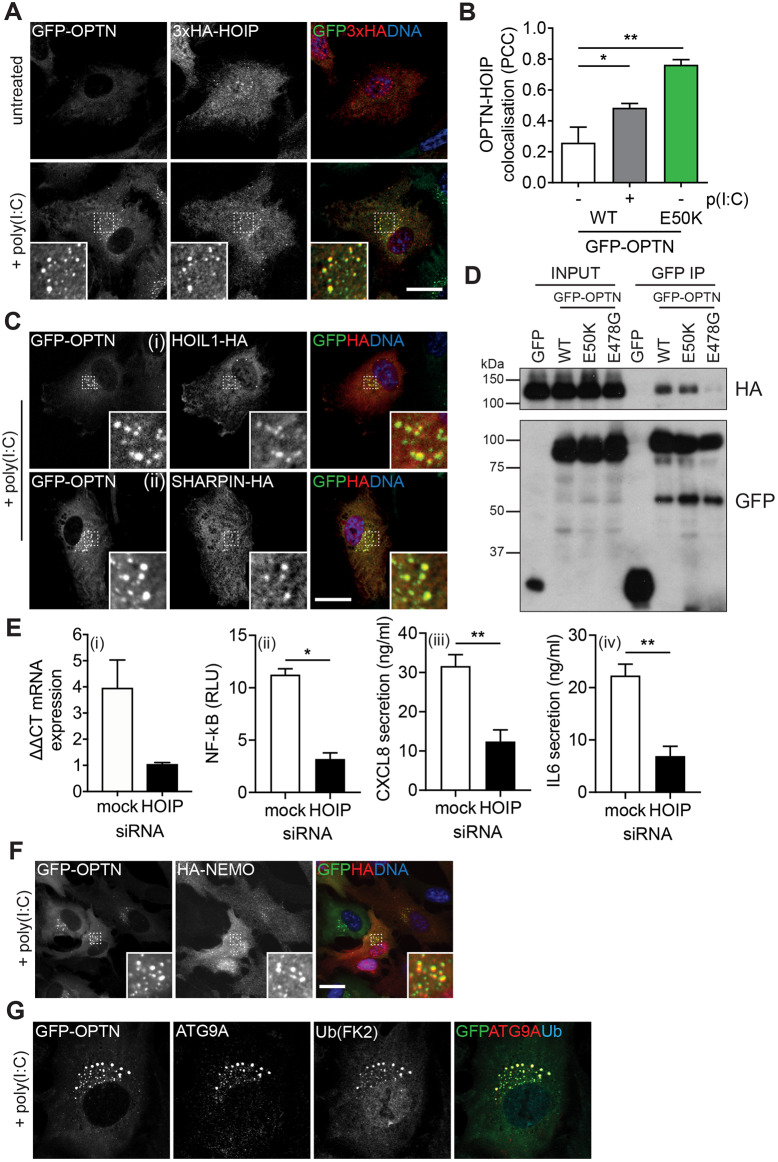
**The LUBAC is recruited to foci.** (A) Confocal microscopy images of RPE cells stably expressing GFP–OPTN (green) and 3×HA–HOIP and treated with poly(I:C) for 0 h (top) and 24 h (bottom). Cells were immunostained with an anti-HA antibody (red) and Hoechst to label DNA (blue). Insets show magnified views of regions indicated by dashed boxes. Scale bar: 20 µm. (B) Pearson's correlation coefficient (PCC) calculated for GFP–OPTN versus HOIP after treatment with poly(I:C) for 0 and 24 h. Bars represent the mean±s.e.m. of *n*=3 independent experiments. Cells were quantified from ≥5 randomly selected fields of view (1–2 cells/image). *P<0.05; **P<0.01 (one-way ANOVA and a Bonferroni post-hoc test). (C) Confocal microscopy images of RPE cells stably expressing GFP–OPTN (green) and HOIL1–HA (top) or SHARPIN–HA (bottom) and treated with poly(I:C) for 24 h. Cells were immunostained with an anti-HA antibody (red) and Hoechst to label DNA (blue). Insets show magnified views of regions indicated by dashed boxes. Scale bar: 20 µm. (D) Immunoblot of GFP immunoprecipitations (IP) from HEK293T transiently transfected with GFP, GFP–OPTN wild-type (WT), E50K and E478G probed with GFP and HA antibodies. Input, 2%. (E) Graphs of HOIP mRNA expression (i), NF-κB luciferase reporter activity (ii) and CXCL8 (iii) and IL6 secretion (iv) in RPE cells transfected with mock or HOIP siRNA and treated with poly(I:C) for 24 h. Bars depict mean±s.e.m. of *n*=3 independent experiments. *P<0.05; **P<0.01 (two-sample *t*-test). RLU, relative light units. (F) Confocal microscopy images of RPE cells stably expressing GFP–OPTN (green) and HA–NEMO. Cells were treated with poly(I:C) for 24 h and immunostained with an anti-HA antibody (red) and Hoechst to label DNA (blue). Insets show magnified views of regions indicated by dashed boxes. Scale bar: 20 µm. (G) Confocal microscopy images of RPE cells stably expressing GFP–OPTN (green) and treated with poly(I:C) for 24 h. Cells were immunostained with anti-ATG9A (red) and anti-ubiquitin (clone FK2; blue) antibodies. Scale bar: 20 µm.

We investigated the role of LUBAC at OPTN foci by assessing recruitment of HOIP to wild-type GFP–OPTN foci. Initially, HOIP showed a low level of colocalisation with OPTN in unstimulated cells; however, poly(I:C) stimulation led to the recruitment of HOIP to GFP–OPTN-positive vesicles and an elevation in colocalisation (Fig. 7A,B). Quantification of the colocalisation demonstrated that unstimulated cells expressing the GFP–OPTN E50K mutant showed much higher HOIP recruitment than wild-type GFP–OPTN even after wild-type GFP–OPTN cells were treated with poly(I:C) (Fig. 7B). Next, we tested whether other components of the LUBAC complex were also recruited to the OPTN-positive foci, and found that, upon TLR3 stimulation, both SHARPIN and HOIL1 showed strong colocalisation (Fig. 7C). To confirm the interaction between OPTN and HOIP, we performed GFP immunoprecipitations from HEK293T cells transiently transfected with wild-type, E50K or E478G GFP–OPTN and HA–HOIP. Wild-type and the E50K GFP–OPTN coimmunoprecipitated HA–HOIP, but GFP–OPTN E478G, which completely lacks foci, failed to do so (Fig. 7D).

We assessed the contribution of HOIP (and LUBAC) to NF-κB signalling upon TLR3 activation in RPE cells. Depletion of HOIP by means of siRNA diminished poly(I:C)-induced NF-κB luciferase activity and secretion of CXCL8 and IL6 (Fig. 7E), confirming that HOIP plays a critical role in NF-κB activation downstream of TLR3 in these cells. This data suggests that OPTN can sequester positive regulators of NF-κB signalling in perinuclear foci.

### OPTN foci formation and stabilisation require ubiquitylation

The presence of LUBAC on OPTN foci implied the presence of linear ubiquitin chains on this compartment. Indeed, the OPTN E478G mutant, which is characterised by its inability to bind ubiquitin (Wild et al., 2011) or HOIP (Fig. 7D), is no longer able form foci (Fig. 4A). To ascertain the role of ubiquitin on this compartment we labelled poly(I:C)-induced OPTN foci with an antibody against ubiquitin (FK2), which recognises a variety of chain types including linear chains (Emmerich and Cohen, 2015). In unstimulated cells, antibody staining was very weak and nuclear (data not shown) but after poly(I:C) treatment the ubiquitin FK2 signal was present on GFP–OPTN- and ATG9A double-positive foci (Fig. 7G). Further triple labelling revealed that OPTN and ATG9A, or OPTN and ubiquitin (FK2)-positive compartments also contained HOIP (Fig. S4A). OPTN has a ubiquitin-binding domain that is homologous to that in NEMO and which binds to linear ubiquitin chains (Nakazawa et al., 2016). We cloned a previously described probe composed of three tandem repeats of the NEMO UBAN domain (RFP–3×UBAN), which shows a 100-fold specificity for M1-linked linkages over other chain types (van Wijk et al., 2012). This probe was recruited to the perinuclear foci upon poly(I:C) stimulation and could be blocked by introduction of the F312A point mutation known to abolish ubiquitin binding (Fig. S4B). The presence of ubiquitin chains, LUBAC, OPTN and the 3×UBAN probe on the foci prompted us to investigate whether NEMO itself was also recruited. Indeed, poly(I:C) treatment of RPE cells stably expressing HA-tagged NEMO triggered its recruitment to OPTN foci (Fig. 7F). The presence of both LUBAC and NEMO on these OPTN-positive foci is highly suggestive of a regulatory role in NF-κB signalling by sequestering these components downstream of TLR3.

Despite the requirement for ubiquitin binding in the recruitment of OPTN as demonstrated by the E478G mutant, siRNA depletion of HOIP had little effect on OPTN relocalisation to foci (Fig. S4C), and suggests that OPTN recruitment is not solely dependent on LUBAC-synthesised linear ubiquitin. As the OPTN UBAN domain is capable of binding to both K63-linked and linear ubiquitin, but not to K48-linked ubiquitin (Nakazawa et al., 2016), we hypothesised other chain types might also be present. Ubiquitin containing a single lysine residue at K63, expressed from a ubiquitin mutant construct, was also present on the foci, indicating they are likely to contain a mixture of both linear and K63 chains (Fig. S4D), and thus it is possible that K63 chains are sufficient for the initial recruitment of OPTN.

### OPTN foci formation correlates with innate immune signalling and cytokine secretion

**Fig. 8.**
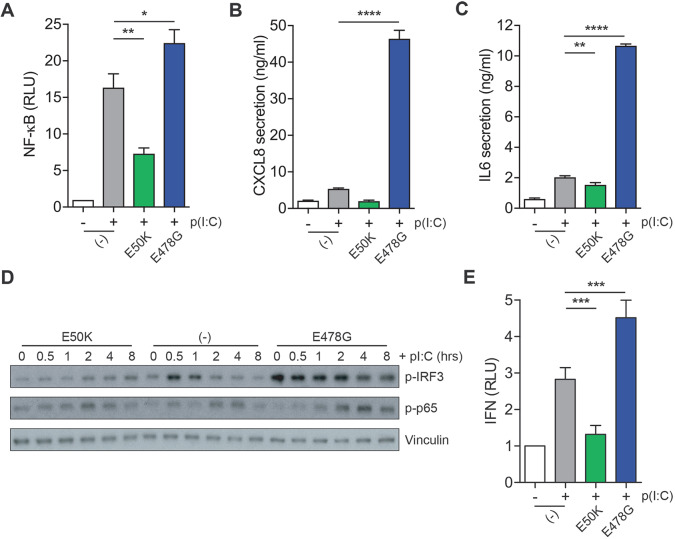
**OPTN mutations regulate innate immune signalling and cytokine secretion.** (A) Relative NF-κB luciferase activity in RPE cells expressing an NF-κB luciferase reporter alone (-), or with coexpression of GFP–OPTN E50K and E478G, and stimulated with poly(I:C) as indicated. Graph depicts mean±s.e.m. of *n*=6 independent experiments. *P<0.05; **P<0.01 (one-way ANOVA and a Bonferroni post-hoc test). (B,C) CXCL8 (B) and IL6 (C) secretion from RPE cells expressing GFP–OPTN E50K and E478G and stimulated with poly(I:C) as indicated. Graphs depict mean±s.e.m. of *n*=6 independent experiments. **P<0.01; ****P<0.0001 (one-way ANOVA and a Bonferroni post-hoc test). (D) Western blots of lysates from RPE cells expressing GFP–OPTN E50K or E478G, stimulated with poly(I:C) and probed with the indicated antibodies. (E) IFNα/β secretory levels from RPE cells coexpressing GFP–OPTN E50K and E478G and stimulated with poly(I:C) for 6 h as determined from luciferase activity induced in the ISRE-reporter cell line 3C11. Graph depicts mean±s.e.m. of *n*=5 independent experiments. ***P<0.001 (one-way ANOVA and a Bonferroni post-hoc test).

The rate of foci formation correlated well with time courses for both the induction of cytokine secretion and the inhibition of NF-κB or IRF3 signalling. In addition, the presence of multiple regulators of NF-κB and IRF3 signalling (LUBAC, NEMO and TBK1) suggested a link between OPTN-induced foci and regulation of these signalling pathways. Previous work has shown that OPTN is a negative regulator of NF-κB and IRF3 signalling, and that ALS mutations or loss of ubiquitin binding perturb these functions (Mankouri et al., 2010; Nakazawa et al., 2016). Therefore, we investigated NF-κB activity in parental RPE cells or RPE cells expressing E50K or E478G and observed a negative correlation between NF-κB activation and OPTN foci formation. Cells expressing GFP–OPTN E50K markedly inhibited NF-κB activity and GFP–OPTN E478G cells showed elevated activity relative to non-expressing control cells (Fig. 8A). Next, we assessed the effect of these mutations on cytokine secretion downstream of NF-κB signalling. RPE cells overexpressing GFP–OPTN E50K showed a reduction in CXCL8 and IL6 secretion, whereas OPTN E478G cells displayed a dramatic increase in secretion of both (Fig. 8B,C). Notably, basal secretion of CXCL8 and IL6 are also elevated in OPTN E478G cells (Fig. S5C,D). These results are consistent with data obtained by immunoblotting (Fig. 8D).

As RIG-I stimulation with pppRNA also induced OPTN foci formation, we next investigated whether OPTN regulated cytokine secretion in this context. As with TLR3 stimulation, the E50K mutant reduced CXCL8 and IL6 secretion in response to pppRNA, while the converse was true for the E478G mutant (Fig. S5A,B). Thus, OPTN appears to regulate the innate immune response to viral RNA generally.

Since OPTN has also been implicated in IRF3 signalling, we next determined the impact of OPTN mutations on this pathway. We investigated the activity of this pathway in mutant cell lines and, again, found that overexpression of the OPTN E50K mutant blunted the IRF3 response, as determined by p-IRF3 immunoblotting, and IFNα and IFNβ (IFNα/β) release assays (Fig. 8D,E). Conversely, the OPTN E478G mutant showed high levels p-IRF3 prior to stimulation, which remained elevated, and a concomitant increase in IFNα/β secretion (Fig. 8D,E). Thus, the propensity to form foci correlates well with NF-κB and IRF3 signalling output and appears to indicate that the formation or presence of foci is refractory to both signalling pathways.

## DISCUSSION

In order to establish an appropriate immune response and prevent chronic inflammation, cells must tightly regulate innate immune signalling and cytokine secretion. The central role of OPTN in negatively regulating these signalling pathways is becoming increasingly clear and different mutations, which modify the ability of OPTN to modify these pathways, appear to lead to distinct diseases. Here, we establish an RPE cell model to investigate the role of OPTN in innate immune signalling. Using this system, we show that OPTN translocates to Golgi-proximal foci in response to exogenous RNA and that this compartment negatively regulates downstream signalling responses. Expression of different disease-causing OPTN mutants leads to either constitutive foci formation in the absence of stimulation, and a concurrent attenuation of IRF3 and NF-κB signalling and cytokine secretion, or the converse.

Our ultrastructural characterisation of the OPTN-positive foci reveals that they are not aggresomes as previously suggested (Mao et al., 2017), but clusters of tightly packed small vesicles of ∼30–40 nm diameter. This vesicle cluster is concentrated in a concise space despite lacking an outer limiting membrane. Our double-labelling experiments suggest that the OPTN foci overlap with ATG9A, a transmembrane protein with a key role in autophagy. ATG9A has a very dynamic trafficking itinerary, cycling between the Golgi complex and the endocytic pathway (Noda, 2017). The exact role of ATG9A remains to be established; however, it has previously been shown to be important during autophagosome biogenesis and maturation (Yamamoto et al., 2012). Our results show only a partial colocalization between ATG9A and LC3, a marker for autophagosomal membranes. This result, although surprising, is supported by previous findings that show that ATG9A only transiently associates with the phagophore initiation site (Orsi et al., 2012; Karanasios et al., 2016). Thus, OPTN might regulate post-Golgi trafficking and sorting of ATG9A-containing vesicles to the phagophore. In addition, as a selective autophagy receptor, OPTN may control the spatiotemporal recruitment of ATG9A vesicles to the site of autophagosome formation. This agrees with the recent finding that autophagy receptors cooperate with TBK1 to recruit the ULK1 complex to initiate autophagosome formation (Vargas et al., 2019). Therefore, the OPTN-positive foci could be a compartment that accumulates post-Golgi trafficking intermediates or marks the site of autophagosome biogenesis.

Our work also highlights the correlation between the formation of OPTN foci and the role of OPTN in negatively regulating NF-κB and IRF3 signalling. Our data demonstrates that OPTN expression is upregulated in poly(I:C)-stimulated RPE cells and occurs with kinetics similar to those of both NF-κB inactivation and OPTN foci formation. Furthermore, we were able to identify and localise several key mediators of NF-κB and IRF3 signalling to OPTN foci, including TBK1, NEMO, CYLD and components of the LUBAC complex. At first sight OPTN foci formation and IRF3 regulation do not seem to correlate, as p-IRF3 and its activator p-TBK1 are maximal during the first 2 h post-poly(I:C) stimulation, which was much earlier than the elevation in visible foci. Data presented here suggests that OPTN migration to the foci is pivotal in the regulation of IRF3 and immune activation. The loss of foci caused by expression of the OPTN E478G mutant resulted in IRF3 hyperactivation, and the opposite was seen with expression of the foci forming OPTN E50K. One possibility is that OPTN may form smaller clusters during the early stages of an immune response that aggregate to form the visible foci at later time points. Nevertheless, if they do form, it seems likely that they require OPTN binding to ubiquitin and the kinase activity of TBK1 to inhibit the immune response, as blocking both results in the hyperactivation of IRF3 downstream of TLR3 activation.

In addition to physically sequestering signalling molecules, the foci could also be involved in actively switching off TLR3 signalling. The foci-resident DUB CYLD has previously been shown to target NEMO and RIPK1, resulting in the inhibition of TNFα-induced NFκB activation in a process dependent on OPTN expression (Nagabhushana et al., 2011). It is possible that this process occurs in the OPTN foci during a TLR3-stimulated immune response. Finally, the presence of both ATG9A and LC3 at some OPTN foci could indicate that autophagy is utilized to regulate the TLR3 immune response, but further work would be needed to support this notion. Taken together, this data suggests a model in which NF-κB signalling generates a negative-feedback mechanism to prevent excessive signalling via upregulation of OPTN expression. We propose that the expression of OPTN is tied to its propensity to oligomerise via dimerisation or tetramerisation or polyubiquitin chain binding leading to foci formation, sequestration of NF-κB or IRF3 signalling machinery and the inhibition of further signalling, possibly via deubiquitylation and autophagy. The OPTN E50K mutant displays a heightened propensity to form oligomers (Li et al., 2016), and this property may explain the observed constitutive foci. Alternatively, the loss of ubiquitin binding seen with the OPTN E478G mutant might prevent foci formation by blocking oligomerisation through polyubiquitin chain binding. Other disease-associated mutations may also alter the ability of OPTN to oligomerise or to recruit proteins into foci and lead to perturbed downstream outputs.

Notably, the OPTN foci described here also show striking similarity to those described in a number of other situations. In particular, activation of the cGAS–STING pathway by cytosolic DNA leads to the trafficking of STING from the ER to an ER-Golgi intermediate compartment (ERGIC), which is also positive for ATG9A (Ishikawa et al., 2009; Saitoh et al., 2009). Trafficking of STING from the ER to this compartment is required for the induction of IRF3 signalling, while ATG9A negatively regulates this process (Dobbs et al., 2015; Saitoh et al., 2009). Recent work has also defined a role for STING in the induction of autophagy in response to cGAMP, cytosolic DNA or DNA viruses, and that the ERGIC serves a membrane source for autophagosome formation in this context (Gui et al., 2019). As an important mediator of autophagy and innate immune signalling, it is tempting to speculate that OPTN might participate in an analogous process in response to exogenous dsRNA or RNA viruses. Other proteins including the NLRP3 inflammasome or OPTN binding partners TRAF3 and TBK1 have also been found to localise to similar Golgi-proximal perinuclear microsomes upon stimulation (Chen and Chen, 2018; Pourcelot et al., 2016; van Zuylen et al., 2012), suggesting that this Golgi-proximal platform might be a common mechanism to regulate signalling, cytokine secretion and autophagy induction in response to diverse PAMPs.

## MATERIALS AND METHODS

### Antibodies, plasmids and reagents

Antibodies used in this study were against: CIMPR (IGF2R) [sc-53146; Santa Cruz Biotechnology; immunofluorescence (IF) 1:50], EEA1 (610457; BD Biosciences; IF 1:100), EF2 (EEF2) [sc-13004; Santa Cruz Biotechnology; western blotting (WB) 1:1000], GFP (A11122; Life Technologies; WB 1:1000), HA (11867423001; Roche; IF 1:400), HA (H9658; Sigma; WB 1:1000), LC3 (M152-3; MBL; IF 1:100), LAMP1 (H4A3; Developmental Studies Hybridoma Bank, University of Iowa; IF 1:100), Myc (05-724; Millipore; WB 1:1000, IF 1:200), OPTN (HPA003360; Sigma; IF 1:100), p-IRF3 (4947; Cell Signaling; WB 1:1000), p-p65 (3033; Cell Signaling; WB 1:1000), p-TBK1 (5483; Cell Signalling; 5483; WB 1:1000, IF 1:100), TGN46 (AHP500; Bio-Rad; IF 1:100), ubiquitin (BML-PW8810; Enzo Life Sciences; IF 1:200), vinculin (MAB3574; Millipore; WB 1:1000) and VTI1A (611220; BD Biosciences; IF 1:100). The ATG9A antibody (ab108338; Abcam; IF 1:100) was a kind gift from Margaret S. Robinson (CIMR, University of Cambridge, UK). Rabbit polyclonal antibodies raised against GFP and MYO6 were generated in-house as described previously (Buss et al., 1998).

Cells were treated with poly(I:C) (Enzo Life Sciences) at 10 µg/ml, LPS (Enzo Life Sciences) at 200 ng/ml, 2′,3′-cGAMP (Invivogen) at 10 µg/ml, Pam3CSK4 (Invivogen) at 10 µg/ml, 5′ triphosphate double-stranded RNA (pppRNA) (Invitrogen) at 500 ng/ml, and BX795 (Sigma) at 500 nM. All treatments were for 24 h unless specified otherwise.

GFP-OPTN pEGFPC2 has been described previously (Sahlender et al., 2005) and was subcloned into the pLXIN retroviral packaging plasmid (Clontech) for stable cell line production. GFP–OPTN E50K and E478G pLXIN mutants were generated by site-directed mutagenesis. The myc-BirA*-OPTN pLXIN vector was created by subcloning OPTN into the myc-BirA* pLXIN plasmid described previously (O'Loughlin et al., 2018). For OPTN-BirA*-HA pLXIN, BirA* was amplified by PCR, introducing a C-terminal HA tag, and inserted into pLXIN. OPTN was subcloned into this vector 5′ to the BirA* tag.

HA-Ub K63 pRK5 and NF-κB-TA-LUC-UBC-GFP-W pHAGE were obtained from Addgene (#17606 and #49343, respectively). NEMO, TLR3, CYLD, SHARPIN, HOIP and RBCK1 were obtained from Addgene (13512, 13641, 15506, 50014, 50015 and 50016, respectively) and subcloned into pLXIN. Full-length IFT74 was generated by Gibson assembly of MGC clones 8322576 and 6614193 (Dharmacon, GE Healthcare). MTMR6 was obtained from Sino Biologicals (HG15192) and the IFI35 open reading frame was synthesised as a Gblock from Integrated DNA technologies. All were subcloned into pLXIN with HA tags.

The CRISPRi lentiviral vector pU6-sgRNA EF1Alpha-puro-T2A-BFP was a kind gift from Luke Gilbert (Department of Urology, University of California San Francisco, CA, USA). Protospacer sequences targeting TLR3 5′-GATTTCATCAGGGAAGTGTG-3′ or a control non-targeting sequence (GAL4) 5′-GAACGACTAGTTAGGCGTGTA-3′ were inserted by restriction cloning.

3xUBAN pRFPC3 was generated as a gBlock (Integrated DNA technologies) comprising the UBAN sequence of NEMO flanked by a 5′ SalI site and 3′ XhoI-BamHI sites. Plasmid DNA was linearised with XhoI and BamHI and ligated with gBlock DNA digested with SalI and BamHI. Complementary SalI and XhoI overhangs were ligated, destroying the restriction sites and leaving a unique XhoI site at the 3′ end of the UBAN open reading frame which could be used in subsequent cloning steps. This process was repeated three times to generate three tandem duplicates of the UBAN sequence.

### Cell lines and transfection

RPE (hTERT RPE-1; ATCC^®^ CRL-4000™) cells were cultured in DMEM/F12-HAM (Sigma) mixed in a 1:1 ratio and supplemented with 10% fetal bovine serum (FBS; Sigma), 2 mM L-glutamine (Sigma), 100 U/ml penicillin and 100 µg/ml streptomycin (Sigma). HEK293T and Phoenix cells (293T; ATCC^®^ CRL-3216^™^ and Phoenix-GP; ATCC^®^ CRL-3215^™^) were cultured in DMEM containing GlutaMAX (Thermo Fisher Scientific) and supplemented with 10% FBS and 100 U/ml penicillin and 100 µg/ml streptomycin.

Stably expressing cell lines were generated using retrovirus or lentivirus produced in the Phoenix retroviral packaging cell line or HEK293T cells, respectively. Cells growing in 100 mm dishes were transfected with 10 µg retroviral transfer vector DNA and 25 µl Lipofectamine 2000 (Thermo Fisher Scientific) or 8 µg lentiviral transfer vector DNA, 8 µg pCMV-dR8.91 and 1 µg pMD2.G packaging plasmids using 48 µl TransIT-LT1 (Mirus). Plasmid DNA was mixed with transfection reagent in Opti-MEM (Thermo Fisher Scientific) and incubated for 30 min before addition to cells. After 48 h, conditioned medium was harvested, filtered and added to the relevant cells. Cells were subsequently selected with 500 µg/ml G418 (Gibco), 1 µg/ml puromycin or sorted by FACS. RPE1 dCas9-KRAB cells were a kind gift from Ron Vale (Department of Cellular and Molecular Pharmacology, University of California San Francisco, CA, USA)). For immunoprecipitation experiments, HEK293T cells were transfected in 100 mm dishes using 8 µg plasmid DNA and 24 µl PEI (Polysciences, Inc). DNA was mixed with PEI in Opti-MEM (Thermo Fisher Scientific), incubated for 20 min and added to cells. For siRNA-mediated gene silencing, RPE cells were transfected with ON-TARGETplus SMARTpool oligonucleotides (Dharmacon, GE Healthcare) targeting MYO6 or HOIP using Oligofectamine (Invitrogen). Cells were transfected on both day 1 and day 3 and assayed on day 5.

For RIG-I stimulation experiments, 1 µg pppRNA (Invivogen) was added to 100 µl LyoVec (Invivogen), incubated for 15 min at RT, transfected into cells at a final concentration of 500 ng/ml, and incubated for 24 h.

### Cytokine assays

Cytokine (IL6 and CXCL8) levels in tissue culture supernatants were determined by ELISA assay (DY206 and DY208; R&D Systems). All assays were performed according to the manufacturer's instructions and read on a CLARIOstar microplate reader (BMG Labtech). ELISA data was normalized to viable cell number determined by MTT assay (Boehringer Ingelheim) or CellTiter-Blue (Promega). IFN levels were determined using a HEK293T IFN reporter cell line (clone 3C11) which was obtained from Prof. Jan Rehwinkel (University of Oxford, UK) (Bridgeman et al., 2015). For the IFN assay, IFN reporter cells were cultured on a 96-well plate with 70 μl DMEM medium overlaid with 30 μl of cell culture supernatant. After 24 h, luciferase expression was quantified using a Pierce™ Firefly Luc One-Step Glow Assay Kit (Thermo Fisher Scientific) according to manufacturer's instructions and read on a FLUOstar Omega microplate reader (BMG Labtech).

### qPCR

Total RNA was harvested using a RNeasy Mini Kit and RNase-free DNase treatment (Qiagen), in accordance with the manufacturer's instructions. RNA (1 μg) was converted to cDNA using oligo d(T) primers and Promega reverse transcription kit. Quantitative real time PCR (qRT-PCR) was performed in duplicate using a SYBR^®^ Green PCR kit (Qiagen) on a Mastercycler^®^ ep realplex (Eppendorf) or Quantstudio 7 flex (Life Technologies). The PCR mix was annealed/extended at 60°C for 60 s, for a total of 40 cycles, then a melting curve was performed. Primers for HOIP were 5′-AGACTGCCTCTTCTACCTGC-3′ and 5′-CTTCGTCCCTGAGCCCATT-3′, TLR3 set 1 5′-TCAACTCAGAAGATTACCAGCCG-3′ and 5′-AGTTCAGTCAAATTCGTGCAGAA-3′, TLR3 set 2 5′-CAAACACAAGCATTCGGAATCTG-3′ and 5′-AAGGAATCGTTACCAACCACATT-3′ and the housekeeper gene peptidylprolyl isomerase A (PPIA) 5′-GTGTTCTTCGACATTGCCGT-3′ and 5′-CCATTATGGCGTGTGAAGTCA-3′ or Actin 5′-GCTACGAGCTGCCTGACG-3′ and 5′-GGCTGGAAGAGTGCCTCA-3′. Relative expression was compared between groups using the ΔΔCt method (Livak and Schmittgen, 2001).

### Cell lysate preparation

Cells were plated in a six-well plate and stimulated at ∼80% confluency. Cells were washed and lysed in RIPA buffer (50 mM Tris-HCl, 150 mM NaCl, 0.5% sodium deoxycholate, 1 mM EDTA, 0.5 mM EGTA, 1% IGEPAL^®^ CA-630 and 0.1% SDS) containing protease inhibitor cocktail (Roche) and PhosSTOP™ (Sigma). Cell lysates were sonicated and clarified at 20,000 ***g*** for 10 min at 4°C. Total protein concentration was measured using a Pierce™ BCA Protein Assay Kit (Thermo Fisher Scientific) and used to normalise sample loading.

### Immunoprecipitation

At 48 h post-transfection, cells were lysed with 1% NP-40 lysis buffer (50 mM Tris-HCl pH 7.5, 150 mM NaCl, 1 mM EDTA, 1% NP-40) containing complete protease inhibitor cocktail (Roche), passed repeatedly through a 25 G needle to homogenise, and clarified by centrifugation at 20,000 ***g*** for 10 min at 4°C. Subsequently, clarified lysates were incubated with 10 µl of GFP-nanobody Affi-gel resin (O'Loughlin et al., 2018) for 3 h with mixing. Beads were washed with 1% NP-40 buffer, then TBS and were eluted in SDS sample loading buffer at 95°C.

### Western blotting

Cell lysates and immunoprecipitations were resolved using precast Novex 4-12% Bis-Tris Midi protein gels (Thermo Fisher Scientific) and transferred to methanol-activated Immobilon-P PVDF membranes (Millipore) using a wet transfer system. Membranes were blocked with 5% BSA (Sigma) or 5% milk in TBS containing 1% Tween-20 and incubated with primary antibody overnight at 4°C. Membranes were subsequently probed with HRP-conjugated secondary antibody and washed; bound antibody was detected using enhanced chemiluminescence (ECL) substrate.

### Immunofluorescence

Cells were grown on sterilised coverslips and fixed in 4% formaldehyde. In the case of structured illumination microscopy experiments, cells were grown on acid-washed, high performance, no. 1.5 (170±5 µm), 18 mm square coverslips (Schott). Post-fixation cells were permeabilised in 0.2% Triton X-100 and blocked with 1% BSA. Coverslips were incubated with primary antibody and then fluorescently labelled secondary antibodies (Molecular Probes). Hoechst 33342 (Thermo Fisher Scientific) was used to visualise DNA and biotin with AlexaFluor^®^568-conjugated streptavidin (Molecular Probes). Images were acquired on a Zeiss Axioimager M1, a Zeiss LSM710 confocal microscope, a Zeiss Elyra PS1 super-resolution microscope or a Thermo Fisher CellInsight CX7 high-content microscope. To measure colocalisation, images from randomly selected fields were background subtracted and manually segmented before calculating the Pearson's correlation coefficient using ImageJ and the coloc2 plugin. Alternatively, confocal images from randomly selected fields of view were automatically thresholded using the Costes et al. method (Costes et al., 2004) before calculating the Pearson's correlation coefficient using Volocity software v6.3 (PerkinElmer). Counts of OPTN puncta were performed using the HCS Studio 3.0 software packaged with the Cell Insight CX7 Microscope and the SpotDetector V4 application. Foci-positive cells were scored manually. All statistical analysis was performed in GraphPad Prism.

### CLEM

Cells were plated on alpha-numeric gridded glass-bottom coverslips (P35G-1.5-14-C-GRID, MatTek, MA, USA) at ∼40–50% confluency and fixed with 2% formaldehyde, 2.5% glutaraldehyde and 0.1 M cacodylate buffer for 30 min at room temperature. The reaction was quenched with 1% sodium borohydride for 20 min and cells were stained with Hoechst 33342 (Thermo Fisher Scientific) before washing with 0.1 M cacodylate. Cells were imaged on an LSM780 confocal microscope (Zeiss) and the coordinates of cells selected for imaging were recorded. After confocal image acquisition, cells were secondarily fixed with 1% osmium tetroxide and 1.5% potassium ferrocyanide before being washed and incubated with 1% tannic acid in 0.1 M cacodylate to enhance membrane contrast. Samples were washed with dH_2_O, dehydrated through an ethanol series (70%, 90%, 100%, and absolute 100%) and infiltrated with epoxy resin (Araldite CY212 mix, Agar Scientific) mixed at 1:1 with propylene oxide for 1 h, before replacement with neat Epoxy resin. Excess resin was removed from the coverslip before pre-baked resin stubs were inverted over coordinates of interest and the resin cured overnight. Stubs were removed from the coverslip by immersing the coverslip in liquid nitrogen. Areas of interest were identified through the alpha-numeric coordinates and 70 nm ultrathin sections were collected using a Diatome diamond knife attached to an ultracut UCT ultramicrotome (Leica). Sections were collected onto piloform-coated slot grids, stained with lead citrate and imaged on a FEI Tecnai Spirit transmission electron microscope at an operating voltage of 80 kV.

### NF-κB luciferase assay

NF-κB luciferase reporter cells were plated onto 24-well plates and, at ∼80% confluency, were stimulated with 10 µg/ml poly(I:C) for 6 h. Cells were washed with PBS, lysed in 100 µl Glo lysis buffer and clarified at 20,000 ***g*** for 10 min. Clarified supernatants were mixed 1:1 with ONE-GLO luciferase reagent (Thermo Fisher Scientific) and luminescence was analysed on a CLARIOstar microplate reader (BMG Labtech). To normalise the data, GFP fluorescence of the clarified supernatant was also determined using the same plate reader.

### Secretomics

RPE cells were cultured in SILAC DMEM/F12 (Thermo Fisher Scientific) supplemented with 10% dialysed FBS (Gibco) and the ‘heavy’ amino acids L-arginine ^13^C_6_^15^N_4_ (147.5 mg/l) and L-lysine ^13^C_6_^15^N_2_ (91.25 mg/l; Cambridge Isotope Laboratories), or equal amounts of ‘light’ arginine and lysine (Sigma). Cells were taken through three passages to ensure complete labelling and plated onto 100 mm dishes. At ∼80% confluency, cells were incubated for 18 h in the presence or absence of 10 µg/ml poly(I:C). Subsequently, cells were washed thoroughly with PBS and serum-free medium and incubated for 6 h in 10 ml serum-free medium containing poly(I:C) or vehicle. Conditioned medium was harvested and clarified at 4000 ***g*** at 4°C. Cell counts were used to normalise loading, and equivalent volumes of heavy and light medium were pooled. The volume of the medium was reduced using low molecular mass spin concentrators (Sartorius) and the samples were resolved ∼1.5 cm into a pre-cast 4–12% Bis-Tris polyacrylamide gel. The lanes were excised, cut into chunks and the proteins reduced, alkylated and digested in-gel. The resulting tryptic peptides were analysed by LC-MSMS using a Q Exactive coupled to an RSLCnano3000 (Thermo Fisher Scientific). Peptides were resolved on a 50 cm EASY-spray column (Thermo Fisher Scientific) with MSMS data acquired in a DDA fashion. Spectra were searched against a *Homo sapiens* Uniprot reference database in the MaxQuant proteomics software package (Cox and Mann, 2008). Cysteine carbamidomethlyation was set as a fixed modification and methionine oxidation and N-terminal acetylation were set as variable modifications. Peptide and protein false discovery rates (FDRs) were set to 0.01, the minimum peptide length was set at seven amino acids and up to two missed cleavages were tolerated. Protein differential abundance was evaluated using the Limma package (Smyth, 2005), within the R programing environment (R core team, 2017). Differences in protein abundances were statistically determined using the Student's *t*-test with variances moderated by Limma's empirical Bayes method. *P*-values were adjusted for multiple testing by the Benjamini Hochberg method (Benjamini and Hochberg, 1995). Gene ontology cellular component enrichment analysis was performed using the PANTHER online web tool (Mi et al., 2017).

### BioID proteomics

BioID experiments were performed as described previously (O'Loughlin et al., 2018). BirA*-OPTN and OPTN-BirA* RPE1 pulldowns were performed in triplicate and duplicate, respectively, alongside a set of five matched BirA*-only RPE1 control pulldowns. OPTN pulldowns were compared against the BirA*-only pulldowns using the online tool at CRAPome.org using the default settings, and a threshold of ≥3 FC-B was established to determine candidate OPTN-interacting proteins. Data were visualised in Cytoscape and merged with protein–protein interaction data mined from MIMIx or IMEx curated databases (Orchard et al., 2007, 2012; Shannon et al., 2003).

## Supplementary Material

Supplementary information

Reviewer comments
