# Supplementary material for: OPTN recruitment to a Golgi-proximal compartment regulates immune signalling and cytokine secretion
Source: J Cell Sci. 2020 Jun 15;133(12):jcs239822. doi: 10.1242/jcs.239822 (PMC7328155; doi:10.1242/jcs.239822)
Supplement: Supplementary information [file joces-133-239822-s1.pdf]

**Table S1 – Differential secretion analysis of RPE1 secretome** -/+ poly(I:C). Gene IDs, log fold-change (FC) and p-value scores are provided.

Click here to Download Table S1

**Table S2 – OPTN BioID data.** Gene IDs, fold-change (FC-B) scores and spectral counts from BirA*-OPTN and BirA* only RPE1 pull downs are provided.

Click here to Download Table S2

**Table S3 – OPTN BioID data.** Gene IDs, fold-change (FC-B) scores and spectral counts from OPTN-BirA* and BirA* only RPE1 pull downs are provided.

Click here to Download Table S3

Journal of Cell Science • Supplementary information

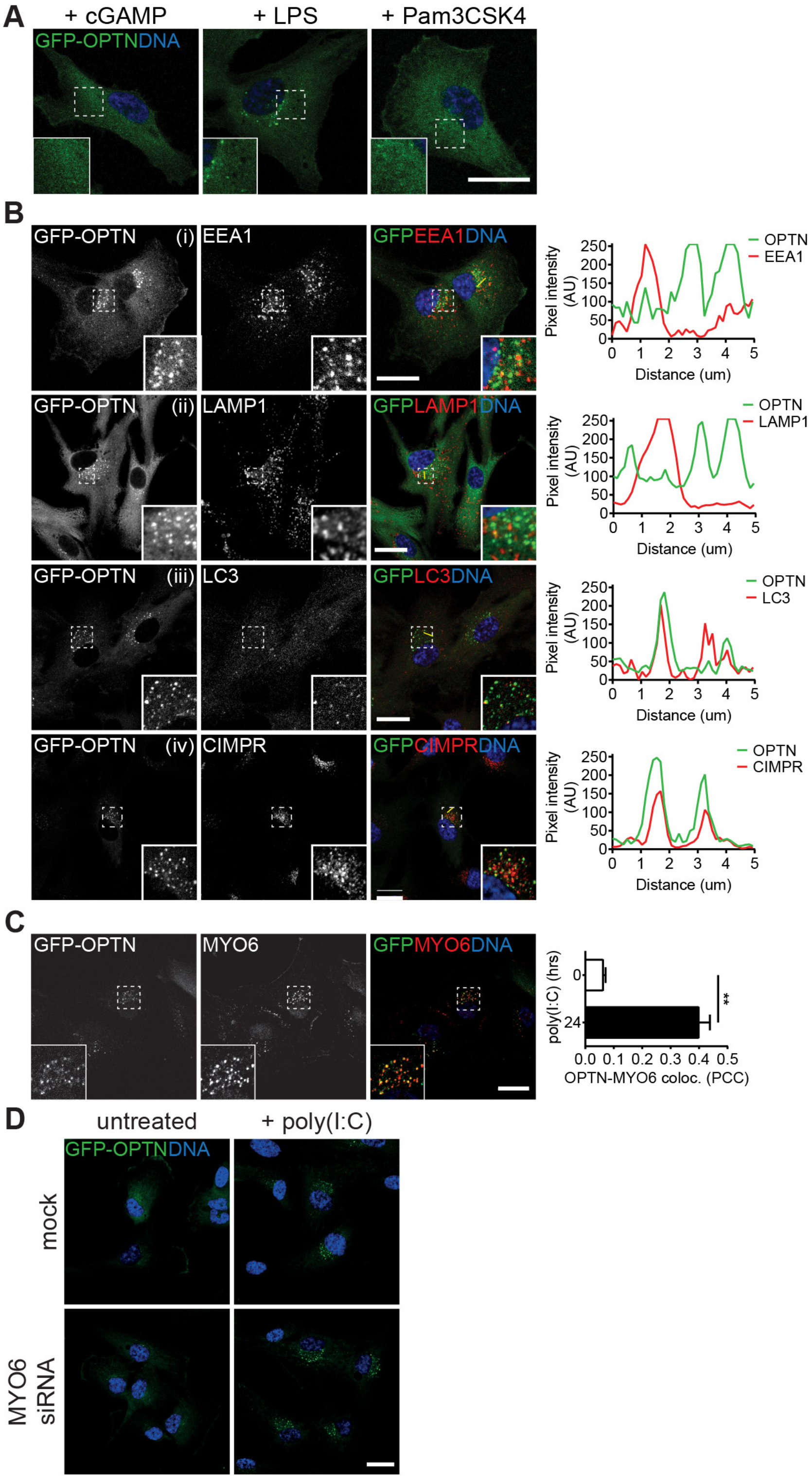


O'Loughlin et al. Figure S1

Journal of Cell Science • Supplementary information

**Figure S1 – Composition of OPTN foci**.
(A) Confocal microscope images of RPE cells stably expressing GFP-OPTN (green) and treated with 2',3'-cGAMP, LPS and Pam3CSK4 for 24 hours. Cells were stained with Hoechst to label DNA (blue). Scale bar, 20 µm. (B) Confocal microscope images of RPE cells stably expressing GFP-OPTN (green) and treated with poly(I:C) for 24 hours. Cells were immunostained with antibodies (red) against EEA1 (i), LAMP1 (ii), LC3 (iii) and CIMPR (iv) and Hoechst was used to visual DNA (blue). Scale bars, 20 µm. Graphs depict pixel intensity in green (OPTN) and red (EEA1, LAMP1, LC3 and CIMPR) channels along line profiles highlighted in image. (C) Confocal microscope images of RPE cells stably expressing GFP-OPTN (green) and treated with poly(I:C) for 24 hours. Cells were immunostained with a MYO6 antibody (red) and Hoechst was used to visual DNA (blue). Scale bar, 20 µm. Graph depicts Pearson's correlation coefficient calculated for GFP-OPTN versus MYO6 after treatment with poly(I:C) for 0 and 24 hours. Bars represent the mean of n=3 independent experiments ±SEM. Cells were quantified from ≥20 randomly selected fields of view (1 cell/image). Statistical significance was calculated using a two-sample t-test. ** = $p<0.01$. (D) Confocal microscope images of RPE cells stably expressing GFP-OPTN (green) and treated with mock (upper panels) or MYO6 (lower panels) siRNA were stimulated with vehicle (left column) or poly(I:C) (right column). DNA was labelled with Hoechst (blue). Scale bar, 20 µm.

Journal of Cell Science • Supplementary information

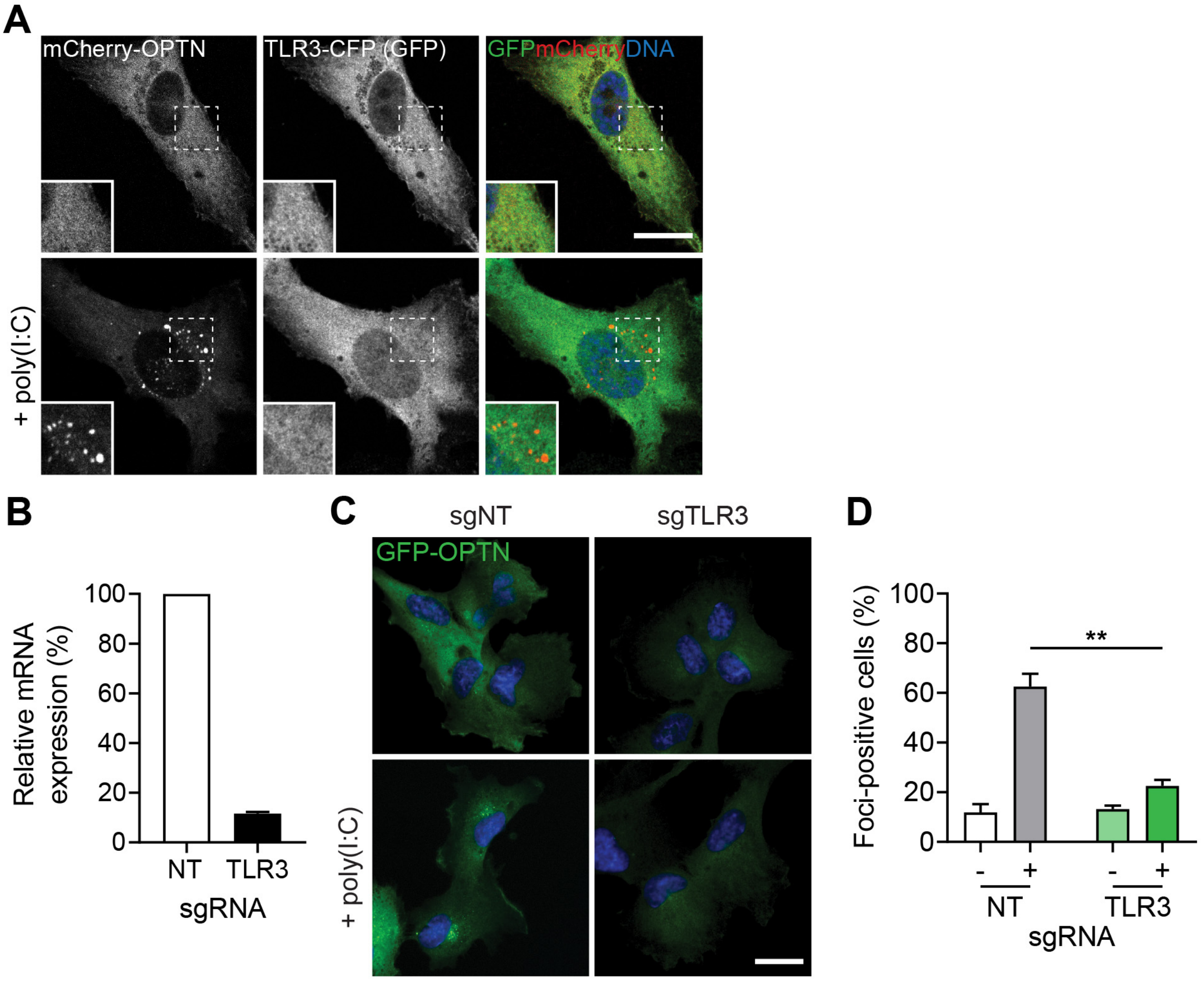


O'Loughlin et al. Figure S2

**Figure S2 – TLR3 knockdown perturbs foci formation.**
(A) Confocal microscope images of RPE cells stably expressing mCherry-OPTN (red) and TLR3-CFP treated with poly(I:C) for 0 hours or 24 hours. Cells were immunostained with a GFP antibody to detect TLR3-CFP (green) and Hoechst to label DNA (blue). Scale bars, 20 µm. (B) Graph depicting relative TLR3 mRNA expression in RPE dCas9-KRAB cells expressing a non-targeting sgRNA (NT) or a sgRNA targeting TLR3. Bar represents the mean from experiments with two different qPCR primers ±SEM. (C) Widefield microscope images of RPE dCas9-KRAB cells stably expressing GFP-OPTN and NT or TLR3 sgRNAs. Cells were treated for 0 hours or 24 hours with poly(I:C) and stained with Hoechst to label DNA (blue). Scale bar, 20 µm. (D) Percentage of NT or TLR3 sgRNA-expressing GFP-OPTN cells containing foci after poly(I:C) treatment. Cells were manually counted from ≥5 randomly selected fields of view across n=3 independent experiments ±SEM. Statistical significance was determined by repeated measures ANOVA and Bonferroni post-hoc test. ** = $p<0.01$.

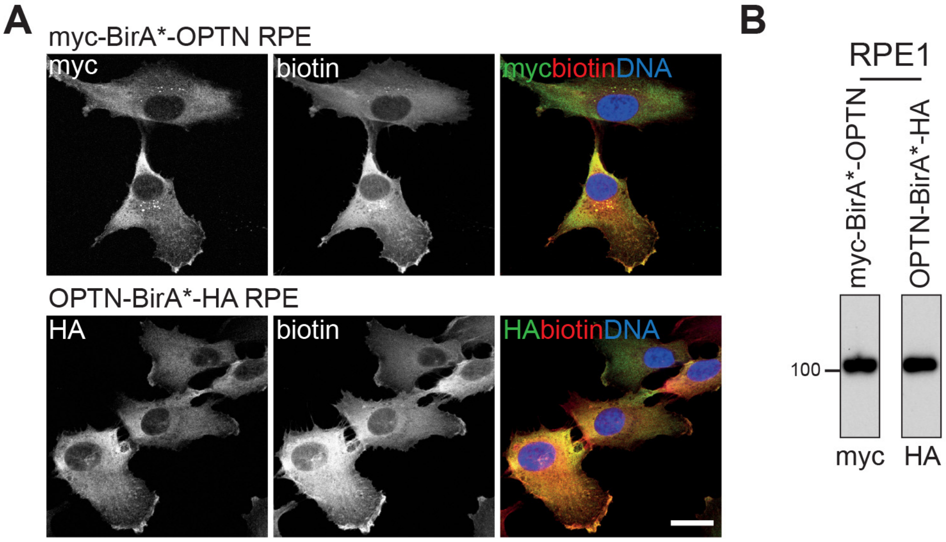


O'Loughlin et al. Figure S3

**Figure S3 – Characterisation of OPTN BioID cells.**
(A) Confocal microscope images of RPE cells stably expressing myc-BirA*-OPTN (upper panels) and OPTN-BirA*-HA (lower panels). Cells were immunostained with an anti-myc antibody (green), biotin was visualised with fluorescently-labelled streptavidin (red) and DNA with Hoechst (blue). Scale bar, 20 μm. (B) Immunoblot analysis of lysates from myc-BirA*-OPTN or OPTN-BirA*-HA RPE cells probed with myc or HA antibodies respectively.

Journal of Cell Science • Supplementary information

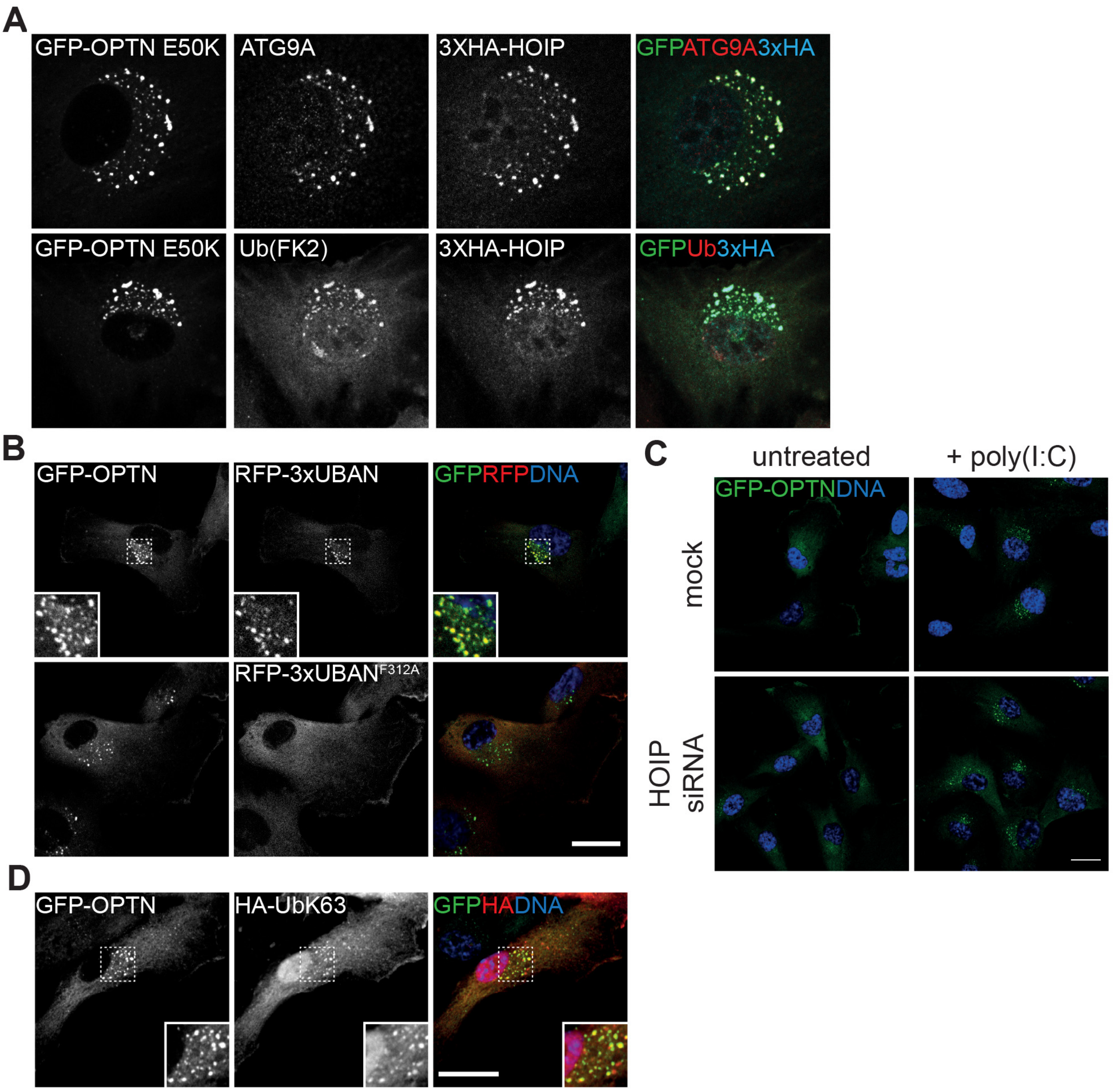


O'Loughlin et al. Figure S4

**Figure S4 – OPTN foci are ubiquitinated but don't require HOIP activity.**
(A) Confocal microscope images of RPE cells stably expressing GFP-OPTN E50K (green) and HA-HOIP (blue) immunostained with anti-ATG9A (red; top panel) or anti-ubiquitin (clone FK2; red; bottom panel) and HA antibodies (blue). Scale bar, 20 µm. (B) Confocal microscope images of RPE cells stably expressing GFP-OPTN (green) and RFP-UBAN (top) and RFP-UBAN F312A (bottom; red) and stimulated with poly(I:C) for 24 hours. Scale bar, 20 µm. (C) Confocal microscope images of RPE cells stably expressing GFP-OPTN (green) and treated with mock or HOIP siRNA were stimulated with vehicle or poly(I:C). DNA was labelled with Hoechst (blue). Scale bar, 20 µm. Mock-treated condition same as shown in Figure S1. (D) Confocal microscope images of RPE cells stably expressing GFP-OPTN (green) and transiently transfected with HA-Ub K63. Cells were treated with poly(I:C) for 24 hours and immunostained with a HA antibody (red) and Hoechst to label DNA (blue). Scale bar, 20 µm.

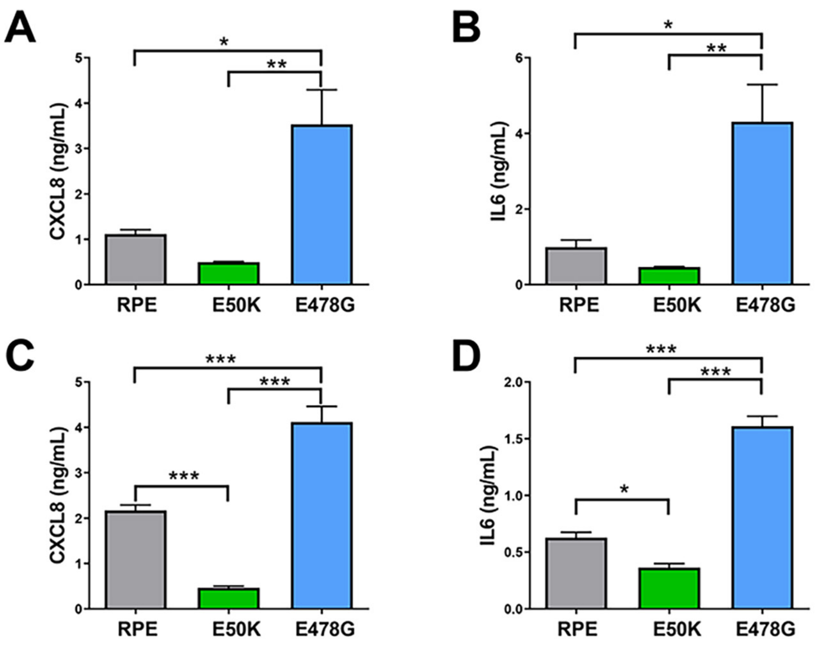


O'Loughlin et al. Fig S5

**Figure S5 – OPTN mutants also modulate RIG-I-induced cytokine secretion and basal cytokine release in RPE cells.**

(A) CXCL8 and (B) IL6 secretion from RPE cells expressing GFP-OPTN wild-type, E50K and E478G and stimulated with the RIG-I ligand pppRNA (10 μg/ml) for 24 hours. Graphs depicts mean of n=3 independent experiments ±sem. (C) CXCL8 and (D) IL6 release from unstimulated RPE cells over a 24 hour period. Graphs depict mean of n=6 ±sem. Statistical significance was calculated by one-way ANOVA and a Bonferroni post-hoc test. * = p<0.05, ** = p<0.01 and *** = p<0.001.
