# Supplementary material for: OPTN recruitment to a Golgi-proximal compartment regulates immune signalling and cytokine secretion
Source: J Cell Sci. 2020 Jun 15;133(12):jcs239822. doi: 10.1242/jcs.239822 (PMC7328155; doi:10.1242/jcs.239822)
Supplement: Reviewer comments [file joces-133-239822_review_history.pdf]

OPTN recruitment to a Golgi-proximal compartment regulates immune signalling and cytokine secretion

Thomas O'Loughlin, Antonina J. Kruppa, Andre L. R. Ribeiro, James R. Edgar, Abdulaziz Ghannam, Andrew M. Smith and Folma Buss
DOI: 10.1242/jcs.239822

Editor: Daniel Billadeau

Review timeline

Original submission:	30 September 2019
Editorial decision:	28 October 2019
First revision received:	19 February 2020
Editorial decision:	11 March 2020
Second revision received:	8 April 2020
Accepted:	14 April 2020

Original submission

First decision letter

MS ID#: JOCES/2019/239822

MS TITLE: OPTN recruitment to an ATG9A-positive compartment regulates immune signalling and cytokine secretion

AUTHORS: Thomas O'Loughlin, Antonina J Kruppa, Andre LR Ribeiro, James R Edgar, Abdulaziz Ghannam, Andrew M Smith, and Folma Buss
ARTICLE TYPE: Research Article

We have now reached a decision on the above manuscript.

To see the reviewers' reports and a copy of this decision letter, please go to: https://submit-jcs.biologists.org and click on the 'Manuscripts with Decisions' queue in the Author Area. (Corresponding author only has access to reviews.)

As you will see, the reviewers raise a number of substantial criticisms that prevent me from accepting the paper at this stage. They suggest, however, that a revised version might prove acceptable, if you can address their concerns. If you think that you can deal satisfactorily with the criticisms on revision, I would be pleased to see a revised manuscript. We would then return it to the reviewers.

Please ensure that you clearly highlight all changes made in the revised manuscript. Please avoid using 'Tracked changes' in Word files as these are lost in PDF conversion.

I should be grateful if you would also provide a point-by-point response detailing how you have dealt with the points raised by the reviewers in the 'Response to Reviewers' box. Please attend to all of the reviewers' comments. If you do not agree with any of their criticisms or suggestions please explain clearly why this is so.

© 2020. Published by The Company of Biologists under the terms of the Creative Commons Attribution License (https://creativecommons.org/licenses/by/4.0/).

Reviewer 1

Advance summary and potential significance to field

Here, the authors have used retinal pigment epithelial cell line RPE1 to study the role of OPTN in immune signalling and cytokine secretion induced by viral RNA. OPTN is known to be a negative regulator of NF-kB and IRF3 signaling in other systems. They show that in response to RNA, OPTN-positive foci are formed in the perinuclear region of the cell. They have characterized these foci by using various markers using microscopy, and also by EM. They suggest that negative regulation by OPTN occurs by recruitment of proteins in these foci. These foci are ATG9A-positive, an interesting and important finding in this study. The E50K mutant of OPTN, which is associated with glaucoma, forms these foci without RNA stimulation and negatively regulates signalling, whereas the E478G mutant, associated with ALS, does not form foci and is not a negative regulator of signalling.

Comments for the author

Most of the data are convincing, but the interpretation of some of the data is not convincing.
I would like the following points to be addressed.

Major points:
1. The data with OPTN mutants suggest a role for OPTN foci in negative regulation of RNA-induced immune signalling. However, time course experiments (Figure 1) show that negative regulation of signalling occurs much earlier than the formation of foci. p-IRF3 levels peak at 30-60 minutes and decline thereafter, p-p65 peaks at 2-4 hours and declines thereafter, IFN secretion peaks at 2-6h, Nf-kB activity peaks at 6h and declines thereafter. OPTN foci form much later at 12 hours and peak levels are reached at 24 hours (Figure 2). There is no increase in foci at 6 hours, and at this time point p-IRF3 level and IFN beta gene expression are already decreased. These data argue against the hypothesis/conclusion that recruitment of proteins to OPTN foci negatively regulates this signalling, which is the main conclusion of this paper. This needs to be discussed.

Minor points:
1. How does OPTN mediate negative regulation of signalling in the foci? The authors suggest that OPTN mediates negative regulation simply by sequestering the active molecules in the foci. I think that the mechanism of negative regulation of signalling in foci by OPTN is likely to be more complex. Decrease in the levels of phosphorylation of p65 and IRF3 are seen with time after the peak levels are attained (Figures 1D, 9D). This indicates that negative regulation by OPTN is an active process. The authors need to consider and discuss other possibilities also. Negative regulation of signalling by OPTN in other systems through recruitment of negative regulators (eg. CYLD) is known.
2. Figure 9. The E478G mutant shows large increase in cytokine secretion upon stimulation of cells with RNA. Does this mutant affect basal levels of cytokine secretion? The basal p-IRF3 level in E478G expressing cells is very high (Figure 9D). It would be appropriate to discuss this point.
3. Source of the RPE1 cell line needs to be mentioned.

Reviewer 2

Advance summary and potential significance to field

O'Loughlin and colleagues here use RPE1 cells as a model to test the function of optineurin (OPTN) downstream of viral infection (mimicked by poly(I:C) exposure). They show that OPTN controls a pro-inflammatory response which is regulated by LUBAC. This regulation results in alterations of signalling from NK-kappaB and IRF3. The complex members of the LUBAC complex, HOIP, HOIL, SHARPIN and ubiquitin are all examined compared to OPTN WT, and E50K or poly(I:C) stimulation. They further examine the role of disease mutations in OPTN (E50K and E478G), and show a nice correlation with the location of OPTN after stimulation or with the mutation to foci which are composed of small vesicular structures. These are seen by CLEM with GFP-OPTN E50K. The authors correlate the colocalization of wild-type OPTN and E50K OPTN with ATG9A to propose that the vesicular cluster seen with CLEM may be positive for ATG9A.

© 2020. Published by The Company of Biologists under the terms of the Creative Commons Attribution License (https://creativecommons.org/licenses/by/4.0/).

The data is clearly presented, and the manuscript is nicely written. The data supports the hypothesis that OPTN translocates to a subcellular compartment which contains key components of the NK-kappaB and IRF3 signalling pathways.
This is an interesting observation. However, the emphasis on ATG9A in the abstract and Discussion seems a bit misplaced, and not enough evidence is presented to confidently conclude ATG9A is present.
Overall the major issues of the paper are
- The use of overexpressed proteins, which should be supported by some endogenous protein work, in particular support for the Bio-ID
- The lack of conclusive colocalization experiments to better define the peri-nuclear compartment, which might have been achieved with triple labelling, for example of GFP-OPTN, HA-interactor (any of those examined) and ATG9A, or Ub.

Comments for the author

While the paper is a nice manuscript it really lacks the data required to connect the morphology with the interactors and firm data to tie in ATG9A. In particular several specific points, major and minor, are listed below.
1. Figure S1B the location of the line scan should be indicated.
2. In Fig. 3 the use of Gal4 is confusing- since it is non-targeting maybe it is better to use “NT”
3. page 8 1st paragraph states BX795 was added for six hours but the legend for Fig. 4 states 8 hours and 16 hours.
4. Figure 5, this is where double labelling CLEM would have been very important.
Also Ub labelling might have been interesting.
5. The OPTN-positive structures in Figure 5A and 5C look different- the ones in 5C are much rounder and larger.
6. Fig. S1B does not show ATG9A with LC3 or any autophagosome marker.
7. Can the data from the “Crapome” be used for any cell type? Despite the different levels of proteins in different cell types?
8. In Fig S2 the control for the BirA constructs. It is not obvious that the peri-nuclear compartment is present of can be induced in this experimental set-up. Indeed it seems as if the Bio-ID was done without stimulation with poly(I:C), which would have been the most appropriate condition.

First revision

<u>Author response to reviewers' comments</u>

<u>Reviewer 1</u>

Most of the data are convincing, but the interpretation of some of the data is not convincing. I would like the following points to be addressed.

Major points:

1. The data with OPTN mutants suggest a role for OPTN foci in negative regulation of RNA- induced immune signalling. However, time course experiments (Figure 1) show that negative regulation of signalling occurs much earlier than the formation of foci. p-IRF3 levels peak at 30- 60 minutes and decline thereafter, p-p65 peaks at 2-4 hours and declines thereafter, IFN secretion peaks at 2-6h, Nf-kB activity peaks at 6h and declines thereafter. OPTN foci form much later at 12 hours and peak levels are reached at 24 hours (Figure 2). There is no increase in foci at 6 hours, and at this time point p-IRF3 level and IFN beta gene expression are already decreased. These data argue against the hypothesis/conclusion that recruitment of proteins to OPTN foci negatively regulates this signalling, which is the main conclusion of this paper. This needs to be discussed.

NFkB activity (p-p65) can be detected between 1-8 hours after poly (I:C) stimulation (Figure 1D) and this activity is lost between 6 and 24 hrs (Figure 1C[iii]) post-stimulation. This change in NFkB

© 2020. Published by The Company of Biologists under the terms of the Creative Commons Attribution License (https://creativecommons.org/licenses/by/4.0/).

activity fits with the emergence of the OPTN foci between 6 and 24 hrs (Figure 2D). LUBAC, a key mediator of NFkB signaling, is recruited to the OPTN foci, and a loss in HOIP expression results in a significant reduction in NFkB activation (Figure 7E[ii]). In RPE cells expressing OPTN mutants we also observe an impact on NFkB activity, with the foci-forming E50K and foci-disrupting E478G mutants inhibiting and elevating NFkB activity, respectively (Figure 8A). Taking all of our data together, we think this provides compelling evidence for an OPTN-based negative regulation of NFkB through the sequestration of LUBAC.

We would agree with the reviewer that the situation regarding the timings between the visible appearance of the OPTN foci and the drop in p-IRF3 levels appears a little less clear. We do know that foci formation and IRF3 phosphorylation are both dependent on TBK-1 activity (Figure 2H, I and Figure below). As with NFkB, the OPTN mutants have a major influence on the activity of IRF3 (Figure 8D and E) and this suggests that the formation of the foci influences this pathway either directly or indirectly. It is possible that OPTN may begin to form foci earlier than we have been able to visualize due to the constraints on the resolution of conventional microscopy or regulate IRF3 signalling by an alternative mechanism. Future experiments will be needed with more advanced microscopy to visualize smaller sub-resolution foci at earlier time points, but this is beyond the scope of this work. In agreement with the reviewer's concerns we have addressed this issue in the discussion.

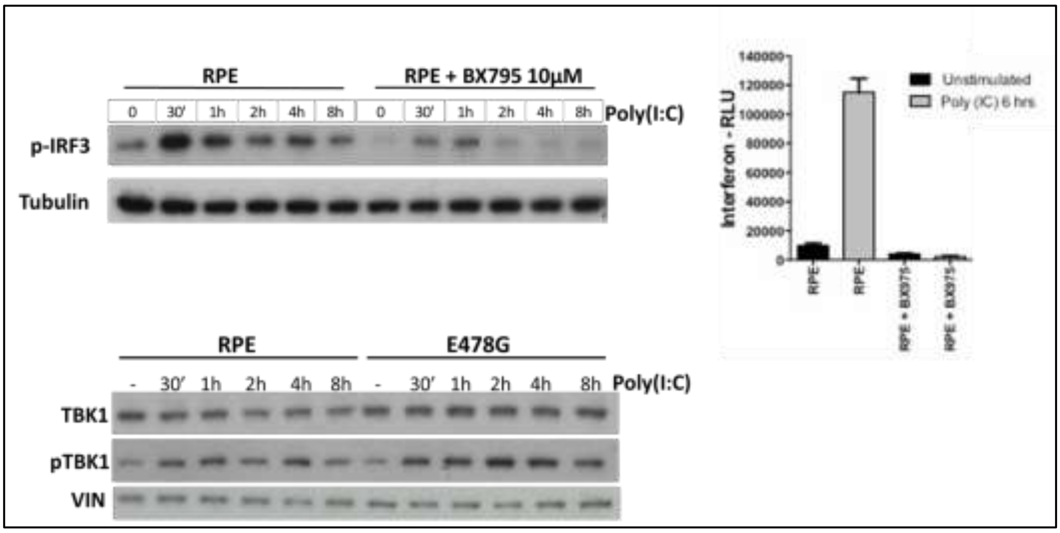

Minor points:

1. How does OPTN mediate negative regulation of signalling in the foci? The authors suggest that OPTN mediates negative regulation simply by sequestering the active molecules in the foci. I think that the mechanism of negative regulation of signalling in foci by OPTN is likely to be more complex. Decrease in the levels of phosphorylation of p65 and IRF3 are seen with time after the peak levels are attained (Figures 1D, 6D). This indicates that negative regulation by OPTN is an active process. The authors need to consider and discuss other possibilities also. Negative regulation of signalling by OPTN in other systems through recruitment of negative regulators (eg. CYLD) is known.

We have now included some additional text into the discussion which describes some alternative mechanisms that could contribute to the OPTN-mediated negative regulation of the signaling:
1. The recruitment of the deubiquitinase CYLD to the foci (Figure 7C[iii]) has previously been shown to inhibit TNF-α-induced NFkB activation and could be involved in the negative regulation of the poly(I:C) response (PLoS One. 2011 Mar 7;6(3):e17477. doi: 10.1371/journal.pone.0017477.)
2. A minority of the OPTN foci also co-stain for LC3 and ATG9A (Figure 5D) after poly(I:C) stimulation, which suggests that some of the signaling molecules could be regulated by autophagolysosomal degradation. The specificity of this process is still unclear and will need further investigation.

© 2020. Published by The Company of Biologists under the terms of the Creative Commons Attribution License (https://creativecommons.org/licenses/by/4.0/).

2. Figure 9. The E478G mutant shows large increase in cytokine secretion upon stimulation of cells with RNA. Does this mutant affect basal levels of cytokine secretion? The basal p-IRF3 level in E478G expressing cells is very high (Figure 9D). It would be appropriate to discuss this point.

We have now included data to address this point (see Figure S5C,D). This data shows that there is a low level of basal cytokine secretion (IL-6 and IL-8) in WT RPE cells and it is elevated in the OPTN E478G mutant cells when compared to control cells.

3. Source of the RPE1 cell line needs to be mentioned

Apologies for this oversight – this has now been clarified in the text.

Reviewer 2

O'Loughlin and colleagues here use RPE1 cells as a model to test the function of optineurin (OPTN) downstream of viral infection (mimicked by poly(I:C) exposure). They show that OPTN controls a pro-inflammatory response which is regulated by LUBAC. This regulation results in alterations of signalling from NK-kappaB and IRF3. The complex members of the LUBAC complex, HOIP, HOIL, SHARPIN and ubiquitin are all examined compared to OPTN WT, and E50K or poly(I:C) stimulation. They further examine the role of disease mutations in OPTN (E50K and E478G),and show a nice correlation with the location of OPTN after stimulation or with the mutation to foci which are composed of small vesicular structures. These are seen by CLEM with GFP-OPTN E50K. The authors correlate the colocalization of wild-type OPTN and E50K OPTN with ATG9A to propose that the vesicular cluster seen with CLEM may be positive for ATG9A. The data is clearly presented, and the manuscript is nicely written. The data supports the hypothesis that OPTN translocates to a subcellular compartment which contains key components of the NK-kappaB and IRF3 signalling pathways. This is an interesting observation. However, the emphasis on ATG9A in the abstract and Discussion seems a bit misplaced, and not enough evidence is presented to confidently conclude ATG9A is present.

We now have included further immunofluorescence data highlighting a very strong colocalization between ATG9A and OPTN (+poly(I:C)) or the mutant OPTN E50K. Our triple labelling experiments further show that the ATG9A and OPTN-positive foci also contain HOIP as well as ubiquitin. We therefore feel that our title stating that OPTN is recruited to an ATG9A-positive compartment is correct. Furthermore, in the abstract ATG9A is only mentioned once in the sentence "These OPTN foci consist of a tight cluster of small membrane vesicles, which are positive for ATG9A", which correctly describes our findings. Finally, we think that it is important to include a detailed discussion of the presence of ATG9A in the OPTN-E50K.

Overall the major issues of the paper are:

1. The use of overexpressed proteins, which should be supported by some endogenous protein work, in particular support for the Bio-ID

The immunofluorescence provided in Figure 6C[i] looks at endogenous (phosphorylated) TBK1. As we pursue HOIP in later work we tried numerous antibodies against this protein but were unable to find an antibody that worked successfully for immunofluorescence. We have also used anti-OPTN, anti-MYO6, anti-Vti1A, anti-ATG9A, anti-LC3 and anti-ubiquitin antibodies that identified endogenous proteins within the cell and foci in Fig. 2, Fig. S1, Fig. 5, Fig. 6, Fig. 7 and Fig. S4.

2. The lack of conclusive colocalization experiments to better define the peri-nuclear compartment, which might have been achieved with triple labelling, for example of GFP-OPTN, HA-interactor (any of those examined) and ATG9A, or Ub.

As suggested by the reviewer we have now included some triple labelled immunofluorescent images with GFP-OPTN WT & E50K vs ATG9A & Ub, ATG9A & HA-HOIP, & Ub & HA-HOIP to better define the peri-nuclear compartment after poly(I:C) or in E50K expressing cells. This data is now included in Figure 5C, Figure 7G and Figure S4.

© 2020. Published by The Company of Biologists under the terms of the Creative Commons Attribution License (https://creativecommons.org/licenses/by/4.0/).

While the paper is a nice manuscript it really lacks the data required to connect the morphology with the interactors and firm data to tie in ATG9A. In particular several specific points, major and minor, are listed below:

1. Figure S1B the location of the line scan should be indicated.

The location of the line scans are indicated in yellow in all cases. These are mostly located within the white boxes that indicate the enlarged area.

2. In Fig. 3 the use of Gal4 is confusing- since it is non-targeting maybe it is better to use "NT"

The labelling has been changed to "non-targeting" as requested.

3. page 8 1st paragraph states BX795 was added for six hours but the legend for Fig. 4 states 8 hours and 16 hours.

Apologies for mislabeling. Cells were treated after 6 hours for the remaining 18 hours of poly(I:C) stimulation.

4. Figure 5, this is where double labelling CLEM would have been very important. Also Ub labelling might have been interesting.

We fully agree with the reviewer's comment here and we are very motivated to conduct a comprehensive characterization of the foci in a future project. However, we feel such a characterization goes beyond the scope of the current work.

5. The OPTN-positive structures in Figure 5A and 5C look different- the ones in 5C are much rounder and larger.

The image in Figure 5A was taken on a widefield microscope, while that in 5C is from a confocal. Even the smaller foci in 5C look the same in the EM images, we simply magnified the larger foci for clarity. We have now included insets magnifying some of the smaller foci that can be seen in the EM. These smaller foci are identical to the larger one and are also comprised of clusters of small vesicles surrounded by small amounts of ER.

6. Fig. S1B does not show ATG9A with LC3 or any autophagosome marker.

We have now included IF with GFP-OPTN WT & E50K vs ATG9A & LC3 -/+ poly(I:C), which shows excellent localization between OPTN and ATG9A but only partial and incomplete localization between OPTN/ATG9A and LC3. Some peripheral foci appear LC3-positive but the specificity of this process is still unclear and will need further investigation.

7. Can the data from the "Crapome" be used for any cell type? Despite the different levels of proteins in different cell types?

Apologies to the reviewer for the miscommunication - no data was taken from the Crapome. We simply used the online tool at Crapome.org to compare our own set of BirA* only RPE1 pull downs against BirA*-OPTN & OPTN-BirA* experiments. We have added some additional text in both the results and materials and methods section to clarify this point.

8. In Fig S2 the control for the BirA constructs. It is not obvious that the peri-nuclear compartment is present of can be induced in this experimental set-up. Indeed, it seems as if the Bio-ID was done without stimulation with poly(I:C), which would have been the most appropriate condition.

The reviewer is completely correct in their interpretation. All experiments were done in the absence of poly(I:C) as these experiments were actually done prior to our characterization of the foci. We retroactively tested identified binding partners for foci localization using GFP-OPTN E50K

© 2020. Published by The Company of Biologists under the terms of the Creative Commons Attribution License (https://creativecommons.org/licenses/by/4.0/).

and identified HOIP as a foci-localised protein. We did perform BioID using RPE cells expressing a BirA*-E50K mutant on one occasion, which generated hits that were very similar to the ones presented in the paper (Figure 7). Due to these findings, we decided not to expand this aspect of the work and to concentrate on characterizing the foci using immunofluorescence and CLEM. We appreciate that in retrospect the ideal experiment would have been to performed the BioID -/+ poly(I:C); however, it would take a considerable amount of time to include this data in the current manuscript and, if the E50K data we have generated is reliable, does not add any additional information.

Second decision letter

MS ID#: JOCES/2019/239822

MS TITLE: OPTN recruitment to an ATG9A-positive compartment regulates immune signalling and cytokine secretion

AUTHORS: Thomas O'Loughlin, Antonina J Kruppa, Andre LR Ribeiro, James R Edgar, Abdulaziz Ghannam, Andrew M Smith, and Folma Buss
ARTICLE TYPE: Research Article

We have now reached a decision on the above manuscript.

To see the reviewers' reports and a copy of this decision letter, please go to: https://submit-jcs.biologists.org and click on the 'Manuscripts with Decisions' queue in the Author Area. (Corresponding author only has access to reviews.)

As you will see, one of the reviewers raises a couple of criticisms that prevent me from accepting the paper at this stage. I don't believe that you will need to perform additional experiments to address their concerns and should be able to address them through editing the title and manuscript. I would be pleased to see a revised manuscript. We would then return it to the reviewers.

Please ensure that you clearly highlight all changes made in the revised manuscript. Please avoid using 'Tracked changes' in Word files as these are lost in PDF conversion.

I should be grateful if you would also provide a point-by-point response detailing how you have dealt with the points raised by the reviewers in the 'Response to Reviewers' box. Please attend to all of the reviewers' comments. If you do not agree with any of their criticisms or suggestions please explain clearly why this is so.

Reviewer 1

Advance summary and potential significance to field

The results presented in this paper suggest that OPTN can inhibit the innate immune response through sequestering key components of NF-κB and IRF3 signalling pathways in a novel perinuclear compartment which is ATG9A-positive. Disease-associated OPTN mutations impact on the formation of the perinuclear compartment and result in hypo- or hyper-activation of the immune response, which could potentially drive the development of a number of human diseases.

Comments for the author

The authors have addressed my concerns satisfactorily in this revised version of the manuscript.

© 2020. Published by The Company of Biologists under the terms of the Creative Commons Attribution License (https://creativecommons.org/licenses/by/4.0/).

Reviewer 2

Advance summary and potential significance to field

Overall the referees comments have been carefully considered and the revisions have been carried out very well.

Comments for the author

There still remain two points of concern they have not adequately addressed.
1) As far as this reviewer can tell, endogenous OPTN has not been shown to colocalize with endogenous ATG9A-positive structures.
2) The structures in Figure 5C are primarily formed by the green GFP-OPTN, and appear to be decorated with ATG9A vesicles. They have no data to show they are are "composed" of ATG9A vesicles, as stated in the figure legend title. This is misleading.

Both points refer to the previous comment about the title. The authors have no evidence to show the structures are composed of ATG9A vesicles, or indeed that OPTN is recruited to the ATG9A-positive compartment. They have only shown overexpressed GFP-OPTN forms a structure and that ATG9A (in vesicles and probably those seen by CLEM) is present at these structures. The title and text should be revised and clarified where appropriate.

Minor point. Figure legend 5 does not have a description of panel D

Second revision

Author response to reviewers' comments

RE: Resubmission of our revised manuscript: JOCES/2019/239822

Dear Prof Billadeau,

We would like to submit our revised manuscript entitled, "OPTN recruitment to a Golgi-proximal compartment regulates immune signalling and cytokine secretion" by Thomas O'Loughlin, Antonina J Kruppa, Andre LR Ribeiro, James Edgar, Abdulaziz Ghannam, Andrew M Smith and Folma Buss for consideration at Journal of Cell Science.

Thank you very much for the positive reviewers' reports and the opportunity to submit a revised manuscript containing changes to the manuscript text. Overall, we are very pleased that both reviewers have acknowledged that "their concerns have been addressed satisfactorily" and "their comments have been carefully considered and the revisions have been carried out very well". To address the final points raised by reviewer 2, we have made the following changes to the text of our manuscript:

Reviewer 2

1. As far as this reviewer can tell, endogenous OPTN has not been shown to colocalize with endogenous ATG9A-positive structures.

Unfortunately, we are not able to perform colocalization experiments of endogenous OPTN and endogenous ATG9A, due to the lack of suitable antibodies. We have, however, performed double labelling experiments using GFP-OPTN and ATG9A as suggested by reviewer 2 in February:
"The lack of conclusive colocalization experiments to better define the peri-nuclear compartment, which might have been achieved with triple labelling, for example of GFP-OPTN, HA-interactor (any

© 2020. Published by The Company of Biologists under the terms of the Creative Commons Attribution License (https://creativecommons.org/licenses/by/4.0/).

of those examined) and ATG9A, or Ub.” This data was included in Figure 5D, Figure 7G and Figure S4.

2. The structures in Figure 5C are primarily formed by the green GFP-OPTN, and appear to be decorated with ATG9A vesicles. They have no data to show they are are “composed” of ATG9A vesicles, as stated in the figure legend title. This is misleading.

The figure legend title for figure 5 has now been changed to “OPTN-positive vesicle clusters colocalise with ATG9A”.

Both points refer to the previous comment about the title. The authors have no evidence to show the structures are composed of ATG9A vesicles, or indeed that OPTN is recruited to the ATG9A-positive compartment. They have only shown overexpressed GFP-OPTN forms a structure and that ATG9A (in vesicles and probably those seen by CLEM) is present at these structures. The title and text should be revised and clarified where appropriate.

The text of the manuscript has been changed accordingly and the title now reads “OPTN recruitment to a Golgi-proximal compartment regulates immune signalling and cytokine secretion”

3. Minor point. Figure legend 5 does not have a description of panel D

We are sorry about this mistake and have amended the figure legend accordingly.

We would like to thank you and the reviewers for your time in assessing our manuscript.
Yours sincerely,

Folma Buss   Thomas O’Loughlin

Third decision letter

MS ID#: JOCES/2019/239822

MS TITLE: OPTN recruitment to a Golgi-proximal compartment regulates immune signalling and cytokine secretion

AUTHORS: Thomas O'Loughlin, Antonina J Kruppa, Andre LR Ribeiro, James R Edgar, Abdulaziz Ghannam, Andrew M Smith, and Folma Buss
ARTICLE TYPE: Research Article

I am happy to tell you that your manuscript has been accepted for publication in Journal of Cell Science, pending standard ethics checks.

© 2020. Published by The Company of Biologists under the terms of the Creative Commons Attribution License (https://creativecommons.org/licenses/by/4.0/).
